# Principles of *in situ* protein sequencing: expansion microscopy-adapted Edman degradation and amino acid recognition

**DOI:** 10.64898/2026.01.29.702630

**Published:** 2026-01-30

**Authors:** Camille M. Mitchell, Sara Z. Tavana, Joanne Z. Peng, Hao Wang, Jiuhan Shi, Chi Zhang, Lilia Evgeniou, Masy Domecillo, Shiwei Wang, Daniel M. Estandian, Alexi G. Choueiri, Evelyn Wong, Sarah Dohadwala, Nicholas F. Polizzi, Laura L. Kiessling, Edward S. Boyden

**Affiliations:** 1 Department of Brain and Cognitive Sciences, Massachusetts Institute of Technology, Cambridge, MA, USA; 2 McGovern Institute for Brain Research, Massachusetts Institute of Technology, Cambridge, MA, USA; 3 Media Arts and Sciences, Massachusetts Institute of Technology, Cambridge, MA, USA; 4 Koch Institute, Massachusetts Institute of Technology, Cambridge, MA, USA; 5 Department of Biological Engineering, Massachusetts Institute of Technology, Cambridge, MA, USA; 6 Center for Neurobiological Engineering, Massachusetts Institute of Technology, Cambridge, MA, USA; 7 K. Lisa Yang Center for Bionics and Yang Tan Collective, Massachusetts Institute of Technology, Cambridge, MA, USA; 8 Howard Hughes Medical Institute, Cambridge, MA, USA; 9 Department of Chemistry, Massachusetts Institute of Technology, Cambridge, MA, USA; 10 Broad Institute of MIT and Harvard, Cambridge, MA, USA; 11 Department of Cancer Biology, Dana-Farber Cancer Institute, Boston, MA 02215, USA.; 12 Department of Biological Chemistry and Molecular Pharmacology, Harvard Medical School, Boston, MA 02215, USA.; 13 The Picower Institute for Learning and Memory, Massachusetts Institute of Technology, Cambridge, MA, USA; 14 Department of Systems Biology, Harvard Medical School, Boston, MA, USA

## Abstract

The ability to map protein identity, with resolution sufficient to infer interactions, would support analysis of how proteins work together, or malfunction, in biological processes and diseases. Although several emerging technologies aim towards single-molecule protein sequencing, they require proteins to be removed from the nanoscale spatial context of cells and tissues. Expansion microscopy (ExM) has facilitated a diversity of chemical analyses by isotropically separating molecules throughout a specimen after permeation via a charged hydrogel, followed by gel swelling. Here, we adapt key protein sequencing steps - Edman degradation and amino acid recognition - to the ExM gel context. Using testbed peptides in ExM gels, we show that N-terminal amino acids can be recognized over multiple cycles of in-gel Edman degradation. These results establish principles of *in situ* protein sequencing and provide a framework for future *in situ* protein sequencing developments, including the development of higher specificity and affinity amino acid binders.

Proteins are central to life processes^1^, and their interactions achieve metabolic or signaling outcomes with great efficacy and precision, in a fashion that depends on nanoscale context^2–4^. As just one example, synaptic NMDA receptor signaling can promote neuronal health, whereas extrasynaptic NMDA receptor signaling can result in neuronal death^5^. A strategy to map all protein identities and positions, within intact cellular contexts, ideally at single molecule resolution (e.g., 1 nm or better) so that interactions can be inferred, could enable the generation of novel hypotheses and reveal insights into how proteins interact to mediate essential and disease-related processes. However, existing spatial protein mapping techniques either do not scale to the entire proteome, and/or fall short of single molecule spatial resolution and sensitivity (see **Supplementary Table 1** for a list of spatial protein mapping strategies and their quantitative properties).

To map all protein identities, locations, and putative interactions, ideally one would be able to identify individual proteins, with single molecule resolution (e.g., ~1 nm or better). To this end, expansion microscopy (ExM) is a tool that involves the chemical anchoring of specific amino acids within a protein to a cell- or tissue-permeating swellable hydrogel; the biological specimen is then chemically softened (e.g., with heat and detergent, or enzymatic, treatment), and then immersed in water, which causes isotropic expansion of the specimen-hydrogel composite^6,7^. The net outcome is that a microscope’s effective resolution is improved by an expansion factor (e.g., ~4.5-fold, in the original version). ExM has been used in hundreds of experimental studies throughout biology^8^. ExM can be iterated, as a sample can be expanded, then a second hydrogel chemically formed in the space opened up by the first expansion, and finally the specimen expanded again, with >50x expansion already having been demonstrated at proof of concept level (by iterating ~4x expansion three times); with novel ExM chemistries that achieve 20-fold expansion in a single step, expansion factors of 100–1000x, or even more, may be feasible^9–11^. The utility of such expansion factors might be justified by a recent study that has shown, by following a specific 10-fold expansion protocol by super-resolution radial fluctuations (SRRF) imaging, that ExM can be used to separate a protein’s proteolyzed fragments from each other with submolecular (e.g., <1 nm) precision and in an isotropic fashion that significantly preserves protein shape^12^. We therefore postulated that ExM could facilitate *in situ* deployment of protein sequencing.

Many efforts are ongoing towards *ex situ* single-molecule protein sequencing. These include using fluorescent N-terminal amino acid binders, or covalent amino acid labels, in conjunction with iterative amino acid removal strategies (e.g. Edman degradation). These technologies operate upon proteins *ex situ*, extracted from the spatial context of intact biological cells and tissues^13–17^. If proteins throughout an expanded cell or tissue were fragmented into pieces, and the pieces separated from each other (Fig. 1A), then one could in principle walk down each peptide fragment, amino acid by amino acid using Edman degradation (Fig. 1B), applying N-terminal binders (or covalent amino acid labels) to such peptide fragments to identify each N-terminal amino acid in turn (Fig. 1C). Finally, computational methods could assemble these sequences into an overall protein identity or even sequence. ExM would support such an effort in at least two ways: it would provide the magnification needed to resolve individual peptide fragments (perhaps assisted by further super-resolution imaging), and would enable peptides to be surrounded by a well-defined chemical environment appropriate for protein sequencing (and to allow them to be more easily followed over the many rounds of imaging required for sequencing, as previously demonstrated for nucleic acids^18–20^).

Edman degradation removes amino acids one at a time using a two-step process. First, phenylisothiocyanate (PITC) reacts with the N-terminal amino acid, and then trifluoroacetic acid (TFA) results in cyclization followed by cleavage^21^, at an efficiency of up to ~99% per round^22^. *Ex situ* single molecule protein sequencing using Edman degradation is an active area of research, e.g., through covalent fluorescent tagging of amino acids^15^, or the application of N-terminal amino acid binders (NAABs)^14,16,23^. Here, we adapted Edman degradation chemistry to ExM to afford an in-gel Edman degradation protocol. We screened through solvents, Edman reagents, and reaction conditions to optimize isothiocyanate (ITC)–peptide conjugation in ExM gels. To optimize the chemical reactions that underpin the process, we analyzed testbed peptides with well-defined sequences in gels, analogous to the path taken by early *in situ* nucleic acid sequencing^24^. We demonstrated that we can evaluate N-terminal amino acid binders as they bind to iteratively exposed N-terminal amino acids of testbed peptides. The creation of such binders is an active area of interest for *ex situ* protein sequencing^14,23,25–31^, and further research in this space will be required for a full protein sequencing protocol to be developed. However, the principles revealed here are fundamental to realizing this objective.

## Results

### Solvent considerations for in-gel Edman degradation

In-gel Edman degradation is a version of Edman degradation that requires anchoring of peptides via some functional groups (akin to solid phase sequencing), for instance through carboxyl groups as performed in *ex situ* protein sequencing^15,32^ or via the primary amine groups, as performed traditionally in ExM^7^. The sequencing of peptides could be performed on neo N-termini generated by selective peptide cleavage after denaturation/digestion, enabling sequencing of many peptides in parallel (see **Supplementary Note 10** and **Supplementary Figure 13c** for modeled exploration of the effect of anchoring and digestion efficiency on the percent of amino acids that are detected). To adapt Edman degradation to the ExM gel, we had to first discover a solvent that was compatible with gels remaining in the expanded state, while enabling PITC conjugation to peptides. The overall chemical reaction architecture for in-gel Edman chemistry is schematized in Fig. 2a (background information on Edman degradation, is in **Supplementary Note 1**, with reagents listed in **Supplementary Table 2**). We cast ExM gels by reacting sodium acrylate, acrylamide, and N,N’-methylenebisacrylamide (BIS, at a slightly high concentration to ensure gel robustness in subsequent steps, resulting in an expansion factor of 3.5x), in a fashion that would either incorporate gel-anchored peptides, or that would omit peptides, as a control. We then cast an acrylamide gel throughout the expanded gel (resulting in a re-embedded ExM gel, or ExMre for short), to stabilize it (at 2.5x expansion, increasing back to 3.5x when exposed to water) and reduce shrinkage under varying solvent and salt compositions (PBS causes ExMre gels to shrink to 2.7x, vs. 1.8x for native ExM gels; Fig. 2b; see **Supplementary Note 2** for information on the ionic strength of these solutions), as used previously for *in situ* RNA sequencing^19^.

To understand how the reaction conditions might influence the gel, we quantified the flat/top side surface of the gels with a ruler, after placing PBS-washed gels in various PITC conjugation solvents for 30 min at 50 °C, followed by adding PITC (30 min at 50 °C). Pyridine and acetonitrile (ACN) are solvents commonly used for PITC conjugation that resulted in gel shrinkage, and in some cases opacity, which could prevent chemical access to the inside of the gel (Fig. 2ci–iii; see **Supplementary Figure 1** and **Supplementary Figure 2** for images and quantifications of gels cited throughout Fig. 2; see **Supplementary Table 4** for full data and statistics for Fig. 2), alone or supplemented with PITC (either at 1:9, or 1:1000, which we also found to suffice; **Supplementary Figure 4**). Many other solvents also turned the gel opaque and/or shrank gels (see **Supplementary Figure 1g** for images). We reasoned that solvents not previously used in Edman degradation, such as dimethylsulfoxide (DMSO) and formamide, might solubilize PITC while being compatible with hydrophilic ExM gels. Such solvents did not cause such severe gel shrinkage (Fig. 2civ–v), alone or with PITC. FITC, a nontraditional Edman reagent that is more water soluble, administered in DMSO with sodium bicarbonate led to no change in gel flat/top surface size (Fig. 2cvi), suggesting these conditions might be useful in the first Edman degradation step. Finally, TFA, needed in the cleavage step, did not shrink ExMre gels (Fig. 2cvii). Thus, we could find solvents compatible with Edman degradation conditions in expanded gel states.

### Testbed peptides anchored throughout ExMre gels for validation of in-gel Edman degradation

Using testbed peptides, we devised a set of mass spectrometry and fluorescent measurements to characterize the efficacy of Edman reagent conjugation and subsequent cleavage. For mass spectrometry, we used a peptide that could be trypsinized from the gel to enable analysis via liquid chromatography coupled with electrospray ionization quadrupole time-of-flight mass spectrometry (or LC/QToF for short) (Fig. 3a; see **Supplementary Figure 12** for discussion of the control peptide). We note that the phenylthiocarbamyl (PTC)-peptide bond resulting from Edman reagent conjugation is labile under preparation conditions for mass spectrometry (see **Supplementary Note 3** for more detail about this limitation), and thus we focus our analysis on the efficacy of ITC conjugation. As expected, conventional Edman solvents that caused gel shrinkage or opacity did not result in good conjugation (see **Supplementary Note 4**). Formamide and DMSO, in contrast, enabled excellent conversion to the PTC-peptide with a conversion rate of ~96% for 1:1000 ratio PITC:formamide (Fig. 3b; see **Supplementary Table 5** for full data and statistics related to Fig. 3) and a conversion rate of >99% for 1:1000 PITC:DMSO (Fig. 3c). FITC in 0.1 M sodium bicarbonate resulted in a lower conversion rate of ~75% (Fig. 3d), as expected from the lower FITC conjugation rate reported in the literature^33^. All three protocols (Fig. 3e), could be conducted without substantial gel shrinkage.

To assess phenylthiohydantoin release, we devised a strain-promoted alkyne-azide cycloaddition (SPAAC) click chemistry fluorescence experiment (Fig. 3f). We used an N-terminal azidolysine peptide that was clicked to a dibenzocyclooctyne (DBCO) fluorophore after various chemical treatments. When the gel was exposed to our Edman degradation conditions, we observed the expected drop in fluorescence due to cleavage (Fig. 3gi–ii), about ~70% of the total (Fig. 3giii; trypsin-mediated positive control). We further explored whether the cleaved phenylthiohydantoin (PTH)-amino acid could be detected directly via LC/QToF (Fig. 3hi), and confirmed PTH-amino acid release under Edman conditions (Fig. 3hii; see **Supplementary Figure 6** and **Supplementary Note 5** for results regarding other amino acids).

### Combining in-gel Edman degradation with bulk N-terminal amino acid detection

We set out to see if an existing N-terminal amino acid binder (NAAB) could detect N-terminal amino acids in the expansion gel. An ideal NAAB would have high affinity and specificity, and a slow rate of dissociation - slow enough to support amplification (e.g., by attaching many fluorophores through hybridization chain reaction (HCR) or enzymatic means^19,34^, which takes minutes to hours) so that ordinary microscopes could be used to identify individual amino acids of individual peptides. Existing NAABs do not yet reach these criteria, with poor affinity, dwell time, and with affinity dependence on the downstream amino acids of the peptide. Making better NAABs is an active area of research^14,23,25–30^, and will likely benefit from efforts in immune and display-based selection, structure based design, and/or AI-driven modeling and design^35^.

We chose as our NAAB *Agrobacterium tumefaciens* ClpS2, engineered for higher specificity towards Phe (ClpS2 St-V1)^25^ (see **Supplementary Note 6** for thoughts on affinity and sequence information). We measured bulk fluorescence throughout the gel upon applying ClpS2 St-V1 with a fused HA tag, followed by addition of a fluorescent anti-HA tag antibody, using ExMre gels equipped with peptides with N-terminal Phe, Trp, Tyr, and Ala (Fig. 4a). We discuss some assumptions and calculations to help interpret images amidst fast binder off kinetics, in **Supplementary Note 7**. We observed high fluorescence for the Phe-bearing peptides, and not the other three (Fig. 4b–d), for instance with an ~8 fold difference comparing Phe to Tyr (for all raw data for Fig. 4 see **Supplementary Table 9**) comparable to our calculated estimate mentioned above (~7 fold difference comparing Phe to Tyr, see **Supplementary Note 8** for calculations). Having established that N-terminal binding was possible, we next sought to test the incorporation of N-terminal binding into a sequencing pipeline. We created gels with peptides bearing Phe on the N-terminus (denoted F_1_ peptide) or in the second position (denoted G_1_F_2_ peptide), so that the NAAB would bind only at the appropriate stage in sequencing (Fig. 4ei–ii). We observed the NAAB to bind F_1_ peptide only in cases when the N-terminal Phe was still remaining (e.g., the DMSO and TFA-only cases), and not when it was occluded (after PITC conjugation) or cleaved (PITC followed by TFA, or the trypsin control) (Fig. 4f). In contrast, for G_1_F_2_ peptide, we only saw NAAB binding when the Phe was exposed after Edman degradation (PITC followed by TFA), and not under conditions where it was occluded by the N-terminal amino acid (DMSO, TFA-only) or by that amino acid bearing PITC, or after trypsin elimination of the peptide (Fig. 4g). In summary, we were able to demonstrate rudimentary protein sequencing using NAABs, over multiple cycles of in-gel Edman degradation. Comparing gel fluorescence for Phe in the 1^st^ vs. 2^nd^ position, we observed a ~50% drop in fluorescence, suggesting a yield comparable to what was estimated from the click chemistry experiment above.

As noted above, single molecule imaging with such a binder would not be straightforward on ordinary microscopes, one of our criteria for technological success. ClpS2 St-V1 exhibits weak affinity (K_d_ of ~1.1 μM) with a dwell time of ~10 seconds^14,23,26^. However, single molecule amplification methods used in ExMre gels for read-out with conventional confocal microscopy, such as hybridization chain reaction (HCR) or rolling circle amplification (RCA), require on the order of hours for signal amplification^19,34^. The binder used here would dissociate before any amplification product could be made. Therefore, this NAAB would not be suitable for single molecule detection in an *in situ* setting.

### Oxidation analysis of amino acid side chains in ExM gels

As proteins being attached to polymerized ExM gels experience a free-radical filled environment, we examined the susceptibility of amino acid side chains to oxidation as a result of free-radical polymerization. The results are described in **Supplementary Note 9**. In summary, we observed a low number of oxidation products for N-terminal Tyr, Phe, Trp, His, Pro and Arg after ExM polymerization, but more substantial oxidation products for both Cys and Met residues as a result of the gelation process (other amino acids, not tested, do not have appropriately reactive side chains). There might be different ways to prevent, reverse, or compensate for such modifications amidst *in situ* protein sequencing; however, one might also be able to guess the identity of a protein even when only some of its amino acids are recognized - and thus a low number of oxidation sites might not hamper the development of a first working *in situ* protein sequencing technology (see **Supplementary Note 10** and **Supplementary Figure 13d** for a simulation of the fraction of correctly identified proteins using a subset of NAABs). Of course, ExM gels that do not involve free radicals are also possible^36,37^.

### In-gel Edman degradation of multiple rounds

We performed assessment of in-gel Edman degradation over 3 cycles, using the LC/QToF-trypsinization assay analysis of embedded peptides, described above, and recording the various peptide species (peptides with up to 3 cleaved N-terminal amino acids, and PTC-peptide from the conjugation step at each round of Edman). Importantly, performing multiple rounds of in-gel Edman degradation provides a way to confirm cleavage of PTC-peptide in the cleavage step of the in-gel Edman degradation reaction, overcoming the mass spectrometry caveat in quantitating the yield of the cleavage reaction in a single round, as noted in **Supplementary Note 3**. In short, formic acid addition, and other sample processing steps that compromise interpretation of PTC-peptide due to simulating the effects of TFA cleavage, occur only at the final step of the procedure, within the instrument itself. Thus such confounds do not compromise the interpretation of PTC-peptide from second and third round of in-gel Edman degradation, which require a fresh N-terminus from a successful cleavage reaction within the gel itself, followed by a successful conjugation, to detect these species. Gel size was relatively constant throughout (Fig. 5a; see **Supplementary Table 6** for all values for Fig. 5), and the products yielded by each step of Edman degradation largely matched what was expected from the 1- and 2-round experiments above (Fig. 5b). In summary, conversion rate of peptide upon reaction with PITC was high each round, with 98–99% conversion (Fig. 5bi, Fig. 5biii, Fig. 5bv). Small amounts of some side products were observed (see **Source Data** for Fig. 5b for all raw chromatogram traces and mass spectra used in Fig. 5), as well as some PTC-peptide from earlier rounds in later rounds of Edman degradation (Fig. 5biv, Fig. 5bvi). These could be the result of reactions involving oxidative desulfuration of the PTC moiety, as reported in the literature^32,38^, or it is possible that some of the novel ingredients used here could result in byproducts (e.g., DMSO and TFA^39,40^), further evidenced by different effects of TFA on gel size after DMSO treatment (Fig. 3eii; Fig. 5a) vs. without DMSO (Fig. 2vii). Perhaps using a different acid (e.g. glacial acetic acid or hydrochloric acid; as previously reported in literature; see **Supplementary Table 2**), temperature, and/or incubation time could further optimize conditions for the ExMre solid-phase conditions. Recent results have also demonstrated the possibility for base-induced Edman degradation^41^.

### An Edman reagent that permits local tethering of an isolated N-terminal amino acid

One concern is that the binding of existing NAABs to an N-terminal amino acid of a peptide is modulated by the second amino acid in the chain (or other downstream amino acids), which could present both chemical and physical hindrance^14,25,26,42^. We thus, over the past several years^42–44^, envisioned a complementary sequencing strategy, with a general workflow as depicted in Figure 6ai, wherein the N-terminal amino acid is first locally tethered to the polymer backbone before being removed from the peptide chain by Edman degradation, for subsequent read-out with an amino acid binder that was designed to bind an isolated amino acid. There would be no second amino acid to interfere with the binder interaction with the first amino acid, and thus perhaps an amino acid binder could have higher affinity and specificity than otherwise possible. As such, ClickP (as we named it), a bifunctional isothiocyanate derivative with an azide group (Figure 6aii), could in principle be used to isolate the N-terminal amino acid of a peptide for later isolated binder interrogation. There could be various ways of implementing this within the gel context, e.g., through initial conjugation of ClickP to the N-terminus of the peptide (i.e., the first step of Edman degradation), followed by local tethering of the N-terminal amino acid to the polymer backbone through click chemistry onto alkyne groups attached to the polymer backbone, followed by cleavage of the amide bond with TFA (Figure 6aiii). The amino acid would then remain bound to the polymer backbone, via click chemistry, in local proximity to its parent peptide. The antigen to be detected would simply be an isolated amino acid, attached to the ClickP group (see Figure 6b).

We here sought to validate whether a binder to such an amino acid could bind to its target in the gel; full realization of a ClickP-based *in situ* sequencing method would require significant further work. We designed binders to target amino acids that have been cleaved off the peptide chain after having been conjugated to ClickP. The ones here were created by Glyphic Biotechnologies (where they are commercially available) - a phenylalanine antibody (denoted F-antibody) and a valine antibody (denoted V-antibody), that target ClickP-tethered and decyclized amino acids (where the PTH-aa form is reverted back into PTC form, exposing the C-terminal carboxyl group, as done previously^45^). The K_d_ values for the F-antibody suggest a dissociation constant of ~75 pM and k_off_ of ~4.5 * 10^−5^ s^−1^, which corresponds to an average dwell time of ~6.2 hours (see **Supplementary Figure 9b**); the V-antibody was similar (see **Supplementary Figure 9a**). These dwell times would, unlike the previous NAABs discussed, support amplification strategies in an *in situ* context, which takes minutes to hours. ClickP showed good conjugation, with a conversion rate of ~99%, and more cleavage after TFA treatment than without, with the caveats acknowledged above regarding interpretation of PTC-amino acid cleavage with mass spec (Fig. 6ci–iii; see **Supplementary Table 7** for all values from Fig. 6c).

ClickP-antigen for various amino acid side chains (comparing Gly, Val, Phe) carrying a biotin group were incubated with separate gels containing anchored streptavidin (Fig. 6d; chemical structures in **Supplementary Figure 10**). Subsequent antibody (F-antibody and V-antibody) binding to the antigens was monitored using bulk fluorescence read-out for both antibodies, as in Fig. 4. The V-antibody showed binding to the valine target over glycine and phenylalanine (Fig. 6ei–iii; see **Supplementary Table 10** for all raw values for Fig. 6e–f). Similarly, the F-antibody showed binding to the phenylalanine target over glycine and valine (Fig. 6fi–iii). Thus, ClickP-amino acid antibodies can bind to their targets in the expansion gel.

### A theoretical assessment of *in situ* protein sequencing

Having established core chemical principles for *in situ* protein sequencing, but with unknowns remaining - particularly when it comes to the affinity and specificity of binders to be invented in the future - we next set out to perform a theoretical assessment of *in situ* protein sequencing, that could guide future developments in the field (details on our strategy and results can be found in **Supplementary Note 10**). In short, the percentage of residues of a protein that remained accessible for read-out through in-gel Edman degradation and N-terminal binding depended on the extent and specificities of fixation, anchoring and digestion chemistries. However, the model suggests that even partial realization of an *in situ* protein sequencing platform could be extremely useful: under the assumptions of our model, with a subset of only 10 amino acid binders, with medium specificity (as defined by our model), 12 rounds of sequencing, with 10% conjugation error, and 30% cleavage error, could correctly identify ~90% of the proteins in the mycoplasma proteome. The human proteome, similarly, can be interrogated quite successfully even with a partially efficacious sequencing scheme.

## Conclusion

We here derive principles of *in situ* protein sequencing. Building from the idea that expansion microscopy (ExM) could decrowd proteins from each other for sequencing, as previously shown for nucleic acids, we demonstrate several chemistries at the proof-of-concept level, including forms of Edman degradation compatible with the ExM hydrogel, and detections of N-terminal amino acids with NAABs. Currently, there are not enough high quality NAABs to support single molecule *in situ* protein sequencing in 3D specimens, but making better ones is an active area of research by many groups. In addition, commercialization efforts are often required to mature a sequencing chemistry (even DNA sequencing required pushes by companies like Illumina to mature to the point of everyday utility in biology). As a result, we focused on bulk measurements of Edman conjugation and degradation in the gel, e.g. with mass spec and bulk gel fluorescence, to evaluate the fundamental chemistries required for *in situ* protein sequencing to take place. Our current results offer a platform for facilitating further *in situ* protein sequencing technology development, e.g. for testing of future NAAB designs, or validating chemical probes for covalent amino acid sidechain labeling in the gel. We showed up to 3 rounds of Edman degradation on peptides in a gel, showing the iterative nature of Edman degradation could be implemented in expansion hydrogels. We also explored whether we could bind isolated amino acids, as might be possible with tethering of them to the gel before cleavage, to reduce steric effects from the downstream amino acids of the peptide.

Several chemical optimizations and validation were required to derive these principles of *in situ* protein sequencing. To enable N-terminal amino acid degradation from peptides embedded in ExMre gels, we screened solvents conventionally employed in Edman degradation chemistry, finding that traditional Edman conjugation solutions led to substantial gel shrinkage and opacity, whereas solvents such as DMSO did not. This preservation was essential for successful Edman degradation to take place. Further work will be required to optimize the yield beyond its current ~70% level, which we speculate to be the result of solvents and reagent interactions with the gel, or potentially interactions with the surrounding gel network during cyclization, unique challenges compared to other Edman degradation platforms.

Existing NAABs have a rate of dissociation that is too fast (~seconds) for fluorescence-based imaging of single molecules via signal amplification schemes (requiring hours), and the second amino acid of the peptide chain can influence binding to the first^25^. Prior work suggests that single amino acid side chains can be determinants of an epitope^46–50^. For instance, the armadillo modular protein, when using a constant peptide for K_d_ ~nM baseline affinity, can exhibit single side chain selectivity^51^. Nevertheless, the relatively small epitope size of a single amino acid side chain, compared to the 4–12 amino acids targeted by many antibodies^52^, and the chemical and physical proximity of subsequent amino acid residues in the peptide chain to the N-terminal amino acid, may make NAAB design challenging. One could imagine using PITC or FITC N-terminally conjugated peptides to raise the baseline affinity of the binders (or even an alternative ITC molecule that is both highly compatible with the hydrogel and provides additional groups for binder attachment), while maintaining specificity for the side chain. Binder design may be significantly enhanced by progress in computational protein design, particularly through AI-driven approaches for modeling and designing protein interactions^53–55^.

We proposed an alternative strategy to overcome the limitations of NAABs, via our heterobifunctional ClickP molecule. This molecule features an isothiocyanate moiety that binds to the N-terminal amine of peptides, along with a click group that can locally tether the amino acid conjugated to the ClickP molecule, e.g. to the hydrogel. We showed this molecule to be compatible with in-gel Edman degradation. In addition, commercially available antibodies produced against these specific antigens had strong affinity, and we demonstrated their specificity to their expected target within the ExMre gels. Local tethering of an amino acid away from its parent peptide chain would prevent influence from subsequent amino acids in the peptide chain and, thus, potentially improve specificity. Future work could include development of the entire workflow combining in-gel Edman degradation with local tethering of N-terminal amino acid with ClickP for a complete cycle of degradation, tethering, and detection of the amino acid from a parent peptide within the polymer network. Other modifications of PITC, e.g. one that allows for DNA oligo conjugation^31^, have been put forth as well.

The ExM protocol itself could be improved in terms of expansion factor, for better decrowding of proteins from each other. Indeed, ExM preserves key aspects of protein shape, enabling imaging at 1 nanometer resolution^12^; future expansion protocols that can expand 100x or 1000x or more, that preserve this high fidelity of expansion, would be particularly valuable. It would also be beneficial to show that such protocols can be applied to proteins in a densely packed, heterogeneous biological sample, such as in a cell or tissue.

Our theoretical assessment of *in situ* protein sequencing, under the assumptions of our model, reveals potential directions for improvement and optimization. Our modeling may guide experimental strategies, such as refining selection parameters and reaction efficiencies to improve peptide fragment retention for subsequent protein identification. Importantly, our model can be modularly modified to test alternative conditions and constraints, and the underlying assumptions can be updated as they become better constrained by empirical data.

## Methods

### Software for figure making

Figure 1a–c; Figure 3a, f, hi; Figure 4a, e; Figure 6a, b, d; **Supplementary Figure 13a-b** were made with BioRender. Figure 2a, b; Figure 6a, b; **Supplementary Figure 8ai-hi; Supplementary Figure 10; Supplementary Figure 11** were made with ChemDraw.

### Synthetic peptide designs

All peptides were ordered from AAPPTEC in aliquots with > 95% purity. 5 mM stock solutions of each peptide were made by diluting lyophilized peptides with UltraPure distilled water, DNase/RNase free (Invitrogen). See **Supplementary Table 3** for exact mass and chemical formulae of all peptides used in the paper, and **Supplementary Figure 11** for structures of all peptides used in the paper.

Edman degradation readouts with liquid chromatography coupled to electrospray ionization quadrupole time-of-flight mass spectrometry (LC-ESI-QToF MS, abbreviated LC/QToF) (results from Figure 3b–d, Figure 5b, Figure 6c) were performed with a peptide that we denoted “A15-peptide” (which stands for AGGAGGLLGGSRGGK{acr}; K{acr} is an abbreviation for lysine modified with an acryloyl functional group). A peptide that we denoted “A9-peptide,” AGGAGK{acr}GLR, was spiked into samples to account for any ionization efficiency fluctuations.

In-gel Edman degradation monitored through bulk fluorescence in Figure 3g was performed with a peptide that we denoted “K{N_3_}15-peptide,” K{N_3_}GGAGGLLGGSRGGK{acr}, where K{N_3_} is an abbreviation for lysine modified with an azide functional group.

Phenylthiohydantoin-phenylalanine (PTH-F) detection was performed with “F_1_” peptide, FGGAGRGLGK{acr} (Figure 3h).

ClpS2 St-V1 bulk fluorescence read-outs comparing different N-terminal amino acids (Fig. 4a) in the ExMre gels were performed with “F_1_” peptide, “W_1_” peptide, WGGAGRGLGK{acr}, “Y_1_” peptide, YGGAGRGLGK{acr}, and “A_1_” peptide, AGGAGRGLGK{acr}. ClpS2 St-V1 bulk fluorescence read-outs combined with Edman degradation chemistry (Fig. 4f–g) were performed with “F_1_” peptide and “G_1_F_2_ ” peptide, GFGAGRGLGK{acr}.

Hemagglutinin-tag (HA) peptide with a K{acr} group (or YPYDVPDYAK{acr}) was used in 9% acrylamide gels in **Supplementary Figure 7b**.

Amino acid oxidation analysis and results (**Supplementary Figure 8a-h**) in ExM gels were performed with XaaGGAGRGLGK{acr} peptides, where Xaa was one of: M, C, W, Y, F, P, H, R. Oxidation analysis, as a result of free-radical polymerization, was performed after trypsin cleavage of the peptides from the gel (or in solution with/without ammonium persulfate (APS; Sigma A3678-25G; we include the catalog number the first time we introduce a chemical in the Methods section) and tetramethylethylenediamine (TEMED; Sigma T7024-25ML)) followed by injection in LC/QToF. Post-translational oxidation modifications, exact mass and chemical formulae, are detailed in **Supplementary Table 3**.

### Making acrylamide, expansion microscopy (ExM), and ExM re-embedded (ExMre) gels

All washes were performed at room temperature (RT), unless otherwise specified (for all sections).

### Acrylamide gel: making the empty gel gelation mixture (i.e. no peptide/protein)

The empty gel gelation mixture (used for gel size change measurements in **Supplementary Figure 2**) was prepared to have a final concentration of 9% acrylamide (w/v) (A9099-25G), 10 mM N,N’-methylenebisacrylamide (abbreviated: BIS; Sigma M7279-25G), 1X PBS (from diluting ThermoFisher 70011 10X PBS pH 7.4), in UltraPure water (Invitrogen 10977015; below, whenever water is mentioned, we use this UltraPure water unless otherwise indicated) to a total volume of 100 μL (see Table 1: **Acrylamide gelation solution)**. Subsequently, pre-chilled 10% (v/v) TEMED and 10% (w/v) APS stock solutions were added to the mix (5 μL each) on ice. Casting the gel was performed as described in section Casting the gel.

### Acrylamide gel: making the gelation mixture with peptide

The peptide-containing gelation mixture (used for **Supplementary Figure 7a-b**) was prepared to have a final concentration of 9% acrylamide (w/v), 10 mM BIS, 1X PBS, 20 μL of peptide (using a stock of 25 mM for **Supplementary Figure 7a**, and using a stock of 5 mM for **Supplementary Figure 7b**), and water to a total volume of 100 μL (final peptide concentration of 5 mM for **Supplementary Figure 7a**, and final concentration of 1 mM for **Supplementary Figure 7b**). Subsequently, pre-chilled 10% (v/v) TEMED and 10% (w/v) APS stock solutions were added to the mix (5 μL each) on ice. Casting the gel was performed as described in section Casting the gel.

### ExM gel: making the empty gel gelation mixture

The empty gel gelation mixture (used for gel size change measurements in Fig. 2c and in **Supplementary Figure 2**) was prepared with 60 μL StockX monomer solution (see Table 2: **StockX monomer solution**), 10 μL 130 mM BIS, and water to a total volume of 90 μL. Subsequently, pre-chilled 10% TEMED (v/v) and 10% APS (w/v) stock solutions were added to the mix (5 μL each) on ice (see Table 3: **ExM empty gel and peptide-containing gelation solutions**). StockX monomer solution was made as follows as in the previously described protocol^7^. Aliquots of StockX monomer solution were kept at −20 °C. Casting the gel was performed as described in section Casting the gel.

### ExM gel: making the gelation mixture with peptide

The peptide-containing gelation mix (used for Fig. 3b–e,g,hii; 4b–d,f–g; 5a–b,f–g; 6c,e–f) was prepared with 20 μL 5 mM peptide of the given sequence, 60 μL StockX, 10 μL 130 mM BIS, with water to a total volume of 90 μL. Subsequently, pre-chilled 10% TEMED v/v and 10% APS w/v stock solutions were added to the mix (5 μL each) on ice (see Table 3: **ExM empty gel and peptide-containing gelation solutions**). Casting the gel was performed as described in section Casting the gel.

### Casting the gel

After gentle mixing, the mixture (taking ~70 μL of solution) was pipetted into a gelation chamber, created using a glass slide (VWR, #48300-026), a 22 × 22 mm #1.5 coverslip (VWR, #48366-227), and one or two layers of parafilm (Uline, S-25929) as spacers, as described previously^56^. Fig. 2c; 3b–e,g; 5a–b, 6c; **Supplementary Figure 1a-h; Supplementary Figure 2a-m; Supplementary Figure 3a-b; Supplementary Figure 4a-c; Supplementary Figure 7a-b; Supplementary Figure 8a-h**, were made with 2 parafilm-thick (~260 μm) chambers (“thick” gel), whereas Figure 3hii; 4b–d,f–g; 6e–f; **Supplementary Figure 6a-b** were made with 1 parafilm-thick (~130 μm) chambers (“thin” gel). By spacing the parafilm stacks 10 mm apart on the glass slide, the final gelation chamber was 10 mm long, 22 mm wide, and with height determined by the parafilm thickness. The gelation chamber was placed within a Tupperware box (with two holes drilled to insert a nozzle for nitrogen purging, and tape to close the holes shut after purging) with a damp paper towel, purged with nitrogen for 5 minutes to remove oxygen, and then incubated at 37 °C for 1.5 hr for gelation. Upon completion of the gelation process, the chamber was disassembled, and the gel was cut into 10 mm × 5 mm rectangles before being transferred to 1X PBS. The gels were washed 3 times in 1X PBS, 5 minutes each.

### Expanding the ExM gels

ExM gels, cut to 10 mm × 5 mm rectangles after gelation, were placed on a glass slide inside a 4 well plate in 1X PBS. 1X PBS was removed and the gels were expanded at RT by washing 3 × 20 min with 5 mL water until they reached ~3.5x expansion. Expanded gels were then cut down to either 5 mm × 5 mm (solvent testing on ExM gels in Fig. 2c, **Supplementary Figure 2a-m**) or used for further re-embedding in Fig. 2c; 3b–e,g,hii; 4b–d,f–g; 5a–b, 6c,e–f.

### Re-embedded ExM (ExMre) gels

Expanded ExM gels (10 mm × 5 mm before expansion, and reaching ~35 mm × ~17 mm in width and length after expansion) were re-embedded following a similar strategy as a previously published protocol^19^. Any remaining water was removed from the 4 well plate, and the gels were submerged in a 5 mL solution with 3% acrylamide (w/v), 0.15% BIS (w/v) obtained from a 1:13 dilution of acrylamide/BIS 19:1, 40% (w/v) solution (AM9024; Fisher Scientific), 5 mM Tris pH 8, 0.075% APS (w/v), 0.075% TEMED (v/v) for 30 min at RT on a shaker (50 rpm). The re-embedding solution was removed from the 4-well plate. A sandwich of 22 × 22 mm #1.5 coverslips (VWR, #48366-227) glued together were placed on either side of the gel. The sandwich consisted of four coverslips for gels with a height of ~700 μm (i.e., the same height as expanded ExM gels made with 2 parafilm thick chambers): in Fig. 2c; 3b–e,g; 5a–b; 6c; **Supplementary Figure 1a-f,h; Supplementary Figure 2a-m; Supplementary Figure 3a-b; Supplementary Figure 4a-c**. The sandwich consisted of two coverslips with a height of ~350 μm (i.e., the same height as expanded ExM gels made with 1 parafilm thick chambers): in Fig. 3hii; 4b–d,f–g; 6e–f; **Supplementary Figure 6a-b**. Then, for all gels, a 50 mm × 24 mm #2 coverslip (VWR, #48382–136) was gently placed on the top of the gel. Gel chambers were placed inside a closed Tupperware box with a damp paper towel (as in section Casting the gel), which was purged with nitrogen for 10 min. Gel chambers were then incubated for 1.5 hr at 37 °C. Expanded re-embedded (ExMre) gels were then cut down to 10 mm × 5 mm (for **Supplementary Figure 3b**) or 5 mm × 5 mm (for Fig. 2c, 3b–e,g,hii, 5a–b, 6c; **Supplementary Figure 1a-f,h; Supplementary Figure 2a-m; Supplementary Figure 3a; Supplementary Figure 4a-c; Supplementary Figure 6a-b**) or 2 mm × 2 mm size gels (for Figure 4b–d,f–g, 6e–f; **Supplementary Figure 7a-b**) for downstream steps.

### Gel flat/top side surface size tests

#### General solvent tests on gel flat/top side surface size

Three separate batches of ExM gels (in expanded state) and ExMre gels, without embedded peptide, were made, cut into ~5 mm × 5 mm squares (~700 μm thick) for solvent testing in Figure 2c. Similarly, 9% acrylamide, ExM (in expanded state) and ExMre gels, without embedded peptide, were cut into ~5 mm × 5 mm squares (~700 μm thick) for solvent testing in **Supplementary Figure 1** (gel images) and **Supplementary Figure 2** (gel size changes). All gels were washed 3 times 10 min washes in 1X PBS. The exact dimensions of each gel were recorded after the 1X PBS washes by measuring the gel flat/top side surface size on a glass slide using a ruler. In cases where the gel tore, the edges were carefully realigned prior to measurement. When the gel folded into a solid form after exposure to the solvents described below and could not be unfolded, measurements were taken from the accessible edges by rotating the vial to obtain the most representative size.

Glass vials (Chemglass, CG-4912–01) were filled with 300 μL of the following organic solvents: trifluoroacetic acid (TFA) (Sigma, T6508-100ML), 1X PBS, water, phenylisothiocyanate (PITC; Sigma, 317861), acetonitrile (ACN) (Sigma Aldrich, 271004), 1:1 ratio pyridine to water (Millipore Sigma, 270407) (all ratios throughout are of volumes added, unless otherwise indicated), dimethylsulfoxide (DMSO) (Millipore Sigma, 276855), formamide (Fisher Scientific, AM9342), 1M Tris pH 8 (AM9856), 1M Tris pH 9.5 (J62084.K2), 0.1 M sodium bicarbonate pH 8.5 (J60408) (Fig. 2cii–vii, and **Supplementary Figure 1bi-ii, ci-ii, di-ii, ei-ii, f**, **h** and **Supplementary Figure 2a-d**). For other experiments, the glass vials were filled with 270 μL of the following organic solvents: ACN, DMSO, formamide, pyridine, 1:1 pyridine:water, triethylamine (Millipore Sigma, 471283), dimethylformamide (DMF) (Millipore Sigma, 270547), 1:1 ratio DMSO to water (**Supplementary Figure 1a, biii, ciii, diii, eiii**, and **Supplementary Figure 2e-l**). The glass vials were filled with 231 μL 0.1 M sodium bicarbonate pH 8.5 for **Supplementary Figure 2m**. Gels were submerged in the solvent or buffer on a heat block at 50 °C for 30 min. For Fig. 2cii–vii, and **Supplementary Figure 1bii, cii, dii, eii**, the solutions were then removed, and the flat/top side surface size of each gel was measured (from the bottom, through the glass vial). Then, PITC or ClickP (Enamine, EN300-37440925) at 1:1000 ratio PITC:solvent or ClickP:solvent, respectively, or fluorescein isothiocyanate (FITC) ‘Isomer 1’ (ThermoFisher, F1906) at a final concentration of 5.9 mM FITC (23:77 of DMSO:0.1 M sodium bicarbonate pH 8.5), was added to fresh solvent or buffer. This solution was added into each glass vial to submerge the gels at 50 °C for 30 min, followed by gel flat/top side surface size measurement again (from the bottom, through the glass vial). For **Supplementary Figure 2e-l**, PITC was added at 1:9 ratio PITC:solvent in the same original solvent (30 μL added to the same 270 μL solvent), and for **Supplementary Figure 2m** FITC in the same original buffer at a final concentration of 5.9 mM FITC (23:77 ratio DMSO:0.1 M sodium bicarbonate pH 8.5; 69 μL of 10 mg/mL FITC in DMSO added to 231 μL buffer) and incubated for 30 min at 50 °C. The gel size was measured again after the solution was removed (from the bottom, through the glass vial).

#### Surface flat/top side size during Edman degradation chemistry

ExMre gels, with embedded peptide, were cut into ~5 mm × 5 mm squares (~700 μm thick) after 3× 10 min 1 mL washes in 1M Tris pH 9.5 at RT in 1.5 mL Eppendorf tubes. The exact dimensions of each gel flat/top side surface size throughout the in-gel Edman degradation were recorded for Fig. 3ei–iii and Fig. 5a. After the 1M Tris pH 9.5 washes, gels were placed on a glass slide and the gel surface size was measured on the glass slide using a ruler. In all the next steps, the gels were submerged in 300 μL of solution and a heat block at 50 °C in glass vials. Gels were transferred to glass vials, washed 2 times with solvent (eg. DMSO or formamide) 5 min each, and gel flat/top side surface size was measured again after removal of the solution from the second wash (measured from the bottom of the glass vial). Then, PITC to solvent (1:1000 ratio, e.g., DMSO or formamide) was added to the gel for 1 hour before removal, followed by the next measurement. The gel was then washed 3 times with solvent (eg. DMSO or formamide) 5 min each before TFA was added for 30 min at 50 °C. After discarding the TFA, the gels were measured again (from the bottom of the glass vial). The gels were washed 2 times with solvent (e.g., DMSO or formamide) 5 min each, and the gel flat/top side surface size was measured again after removal of the last wash. Then, the gels were washed 3 times with Tris pH 8 (Fig. 3ei–iii), 10 min each at RT, measured, and transferred to 1.5 mL plastic Eppendorf tubes. For Fig. 5a, the gels were washed 3 times with 1M Tris buffer pH 9.5, 10 min each at RT, measured, before the next cycle of in-gel Edman degradation. During the two subsequent rounds of in-gel Edman degradation for Fig. 5a the flat/top side surface size was similarly measured.

### In-gel Edman degradation

In-gel Edman degradation was performed as outlined in the schematic in Fig. 3a. First, a heating block in a chemical hood was preheated to 50 °C. Gels were washed in 1.5 mL Eppendorf tubes, 3 times 5 min with 1 mL of 1M Tris pH 9.5 at RT. The gel samples were carefully transferred into glass vials with solvent on the heat block. In all the next steps, the gels were submerged in 300 μL of solution in glass vials that were placed on a 50 °C heat block. Samples were washed with solvent or buffer (e.g., DMSO for Fig. 3c, formamide for Fig. 3b, 0.1 M sodium bicarbonate pH 8.5 for Fig. 3d) two times, 5 minutes each. Samples were then incubated with PITC to solvent (1:1000 ratio) for a duration of 1 hour (Fig. 4f–g, **Supplementary Figure 3b** performed with 1:9 of PITC:solvent; **Supplementary Figure 4a** with 1:100 ratio of PITC:solvent; **Supplementary Figure 4c** with 1:10,000 ratio of PITC:solvent; Fig. 6c performed with 1:1000 ratio ClickP:DMSO). For FITC conjugation (Fig. 3d), 5.9 mM FITC in 23:77 of DMSO:0.1 M sodium bicarbonate pH 8.5 was added to the samples for a duration of 2 hours, in the dark. Following this, samples were washed 3 times 5 minutes in solvent or buffer (except **Supplementary Figure 3b** which was performed with 1 solvent wash; Fig. 4f–g was performed with 2 solvent washes). After removal of the solvent, gels were submerged in TFA for 30 min. TFA was removed and samples were washed with solvent twice, 5 minutes each (this step was omitted for PTH detection in Fig. 3h, see **Edman degradation and PTH detection** methods section). Glass vials were discarded, and gels were placed back into Eppendorf tubes at RT. If only 1 in-gel Edman degradation round was performed, as in Fig. 3b–d, g; Fig. 4f–g; Fig. 6c, the tubes were washed 3 times 10 min each, with 1 mL of 1M Tris buffer pH 8 at RT before trypsinization. If another round of in-gel Edman degradation was performed, as in Fig. 5a–b, the tubes were filled with 1ml of 1M Tris buffer (pH 9.5), and washed 3 times 10 min each at RT, and the pH was checked by immersing pH strips in the buffer solution after the last wash. (Other gels that served as controls with 1 or 2 rounds of in-gel Edman degradation during the multiround experiment were washed 3 times 10 min with 1 mL of 1M Tris buffer pH 8 and stored at 4 °C.) After the last round was performed, the tubes were washed 3 times 10 min with 1 mL of 1M Tris buffer pH 8 at RT before trypsinization.

### LC/QToF analysis of synthetic peptides in the gel

#### Gel preparation

Edman degradation read-outs with LC/QToF were performed with A15-peptide in ExMre gels. Each gel size was 5 mm × 5 mm × ~700 μm after re-embedding (see section: Methods: Re-embedded ExM (ExMre) gels).

#### Trypsinization of peptides from the gels

Peptides were trypsinized from the gels after in-gel Edman degradation (see Fig. 3a; right hand side). After the 3 times 10 min 1 mL 1M Tris buffer (pH 8) washes, they were submerged in 50 μL of 20 μg/mL trypsin (New England Biolabs, #P8101S) in 50 mM Tris-HCl pH 8 in the bottom of the 1.5 mL Eppendorf tube (ensuring they were completely submerged in trypsin solution). The gels were then incubated overnight (~16 hours) at 37 °C. The supernatant (~50 μL) was placed in a mass-spectrometry vial (ThermoFisher, 6PSV9-1P), with the glass insert (ThermoFisher, 6PME03C1SP) and screwed shut (ThermoFisher, 6PSC9ST101X).

The A9-peptide was spiked into all the samples, during trypsinization, at a 5 μM final concentration (the A9-peptide was added along with the 20 μg/mL trypsin for the overnight, ~16 hours, incubation at 37 °C) to monitor any fluctuations in ionization efficiency of the samples (the abundance of A9-peptide from experiments in Fig. 3b–d; Fig. 5b; Fig. 6c were plotted in **Supplementary Figure 12**).

#### Mass spectrometry (LC/QToF)

Samples were brought to the Department of Chemistry Instrumentation Facility (DCIF) at MIT for injection into the LC/QToF. This instrument was a high-resolution Agilent 6545 mass spectrometer coupled to an Agilent Infinity 1260 LC system running a Jet Stream ESI source (mass accuracy of 1–3 ppm using real-time calibration, with a mass resolving power of 45,000 (FWHM) at m/z of 2722; measurable m/z range from 50 to 10,000). The column used was a reversed-phase ZORBAX Eclipse AAA, 3.0 × 150 mm, 3.5 μm, C18 (Agilent part number: 961400-302). For **Supplementary Figure 3b**, a reverse-phase ZORBAX StableBond 300 C3, 2.1 × 150 mm, 5 μm column was used (Agilent part number: 883750-909). Before each run, the instrument was positively tuned, and 10 μL blank water washes were used to wash the column twice. 4 μL of the trypsinized supernatant was injected into the mass spectrometer for ExMre gels with 5 × 5 mm top/flat surface size in Fig. 3b–d; Fig. 5b; Fig. 6c; **Supplementary Figure 3a**; **Supplementary Figure 4a-c;** and for the control curve in **Supplementary Figure 5** (with an estimated concentration of ~50 μM peptide in ExMre gels, ~50 pmol (~50 ng) of trypsinized peptides were injected). 2 μL of the trypsinized supernatant was injected into the mass spectrometer for ExMre gels with 10 × 5 mm top/flat surface size in **Supplementary Figure 3b**. 1 μL of the trypsinized supernatant was injected into the mass spectrometer for non-expanded ExM gels with 10 × 5 mm top/flat surface size for oxidation analysis in **Supplementary Figure 8**. 10 μL of the supernatant was injected into the mass spectrometer for PTH-F detection in Fig. 3hii. The samples were injected using LC Method, which details the mobile phase during the ~23 min estimated run time.

#### LC Method: chromatographic separation method (LC/QToF):

**Table:** 

Time (min)	Solvent A (%)	Solvent B (%)	Flow (mL/min)	Max Pressure Limit (bar)
0.00	99.5	0.5	0.300	400.00
1.50	99.5	0.5	0.300	400.00
15.00	40.0	60.0	0.300	400.00
18.00	5.0	95.0	0.300	400.00
19.00	5.0	95.0	0.300	400.00
20.00	99.0	1.0	0.300	400.00

From 0.00 to 1.50 min, the eluate was sent to waste and not to the Q-ToF. The Q-ToF was turned on at 1.50 min. Solvent A: 0.1% formic acid in water. Solvent B: 0.1% formic acid in ACN.

#### Analysis of LC/QToF data

Bar graphs representing the relative abundance (arbitrary units, a.u.) of different peptide ion species from the LC/QToF were obtained from the area under the curve (AUC) of the chromatogram based on the exact mass of the various species (Agilent MassHunter Qualitative Analysis software). The relative abundance of the ion species was reported throughout the Edman degradation process in the gel in the various conditions.

Specifically, to obtain the AUC in an automated fashion (Fig. 3b–d, Fig. 5b, Fig. 6c, **Supplementary Figure 3a-b, Supplementary Figure 4a-c, Supplementary Figure 5** and **Supplementary Figure 12**), Agilent MassHunter Qualitative Analysis software was opened with the files to analyze. Using the “Compound Analysis” tab, with “Find by Formula”, the given formula and exact mass (see **Supplementary Table 3**) was used to extract the information about each species of interest from the total ion chromatograms (TICs). They were saved as an excel spreadsheet for downstream analysis and plotting. Plots were then made in python (see **Code**).

The results from this search were manually inspected to ensure correct peak identification. The raw TIC, and the corresponding mass spectra of the species of interest were exported as metafiles (‘.emf’) (available in **Source Data**, with an arrow pointing towards retention time and peak of interest).

For Figure 3hii and **Supplementary Figure 8a-h**, the AUC for the species of interest were obtained by manual integration by right clicking the chromatogram and selecting “Extract chromatogram”. In the pop-up window, the mass of the [M+H]^+^ species was inputted, and the detected peak(s) from this extraction were integrated into a single value (the integration considers an error of ± 20 ppm (Δm) : Δm = (ppm error × exact mass) / 1,000,000 for detection).

#### In-gel Edman degradation with PTH detection

F_1_ peptide (3 separate vials received from AAPPTEC), were cast in ExM gels with a peptide concentration of 1mM in the gelation solution, and ~130 μm (i.e., 1 parafilm) thick gelation chamber (see section Methods: ExM gel: making the gelation mixture with peptide and Re-embedded ExM (ExMre) gels). After they were re-embedded into ExMre gels, they were washed 3x with 1X PBS 10 min, and cut into 5 × 5 mm squares on a glass slide. In-gel Edman degradation was performed with PITC to DMSO (1:1000 ratio PITC:DMSO), as described in section In-gel Edman degradation, with some modifications detailed here. Positive control included both PITC and TFA treatments, whereas negative control omitted the PITC. After the 30 min TFA incubation at 50 °C, the 300 μL of TFA was removed, and the gel was directly resuspended in 50 μL of 1:1 ACN to water. After shaking the glass vial 10 times, the 50 μL supernatant was pipetted up and down 20 times. The gel shrank and became slightly opaque in this process. The supernatant, containing the PTH-aa, was placed in a mass spec vial for downstream analysis, where 10 μL of the sample was injected into the LC/QToF (an estimated 2 pmol was injected, if we assume a ~70% Edman efficiency). Analysis of PTH-F abundance was performed using PTH-F exact mass: 282.0827 ± 0.0056 Da (using an error of ± 20 ppm (Δm) : Δm = (ppm error × exact mass) / 1,000,000). Positive control of PTH-phenylalanine, in **Supplementary Figure 6b**, was purchased from TCI, P0367. A stock concentration of 12.5 mM in 1:1 ACN to water (volume ratio, used throughout unless otherwise indicated) with 0.1% TFA was made and stored at −20 °C in 100 μL aliquots. The stock solution was diluted to 100 μM in 1:1 ACN to water and 10 μL of this sample was injected into the LC/QToF (~1 nmol).

#### Control curve for calculating yield on LC/QToF

Peptide sequences were the fragments from A15 peptide expected after its trypsinization from the gel (fragments recovered in Fig. 3b–d): AGGAGGLLGGSR (non-modified peptide), and GGAGGLLGGSR (peptide with cleaved N-terminal amino acid). These sequences were ordered in 3 different 0.8 mg peptide aliquots of each peptide from AAPPTEC (> 95% purity). Peptides were prepared together in solution at various concentrations from 5 μM to 60 μM for the 3 separate replicates for each concentration (“C_product” in **Supplementary Figure 5**: 5, 10, 20, 30, 40, 50, 60 μM, and “I_product” was the abundance of the peptide). In addition, in each solution, a control peptide, namely the A9-peptide, was spiked at 5 μM final concentration in all triplicate samples for each concentration and was used as the standard (“C_standard” was 5 μM, and “I_standard” was the abundance of the A9-peptide). Peptide abundances were analyzed on Agilent MassHunter Qualitative Analysis software. The method for **Analysis of LC/QToF data** was performed for those species to obtain the AUC in an automated fashion.

#### Strain-promoted alkyne-azide cycloaddition (SPAAC) read-out of in-gel Edman degradation

Edman degradation followed by SPAAC for bulk fluorescence read-outs (Fig. 3f–g) were performed with K{N_3_}15-peptide. The ExM gelation solution containing peptides at 1 mM concentration was cast in a gelation chamber with thickness ~260 μm (i.e., 2 parafilm thickness), expanded and re-embedded to reach ~2.7X expansion factor in 1X PBS. Thus, the final peptide concentration in the gels reached ~50 μM. ExMre gels were cut to 5 × 5 mm and reached a thickness of ~700 μm with expansion, with each gel containing ~900 pmol peptide.

The steps were the same as the protocol written in the section In-gel Edman degradation, but with K{N_3_}15-peptide, and with modifications detailed here. As in the in-gel Edman degradation, PITC to DMSO (1:1000 ratio PITC:DMSO) was used for the conjugation step and after removing TFA, the gels were washed 3 times, 10 min each, with 1 mL of 1M Tris buffer pH 8 in 1.5 mL Eppendorf tubes. The gels with condition “PITC:DMSO+TFA+trypsin” were then submerged in 50 μL of 20 μg/mL trypsin in 50 mM Tris-HCl pH 8 in the bottom of the 1.5 mL Eppendorf tube (ensuring they were completely submerged in trypsin solution). The gels for other conditions were submerged in 50 μL of 50 mM Tris-HCl pH 8 (without trypsin). The gels were then incubated overnight (~16 hours) at 37 °C. Then, they were washed in 1X PBS 3 times, 10 minutes each, at RT. 20 μg/mL of DBCO-AF488 (Lumiprobe, #218F0) was prepared, from a stock of 10 mg/mL fluorophore in DMF stored at −20 °C, in 1X PBS (500 μL per tube). Gels were submerged in 500 μL of the solution and checked to be well suspended within the Eppendorf tube (not stuck to the edge throughout the staining process). The click reaction was left to react overnight (~16 hours) at 4 °C, and washed 4 times the next day with 1X PBS, 10 min per wash prior to imaging.

The yield calculation based on the SPAAC assay was defined as:

%yield=(“1”-“4”)/(“1”-“5”)

where the numbers represent the separate conditions as indicated in Fig. 3g: where (1) DMSO only, (2) PITC to DMSO (1:1000 ratio PITC:DMSO), (3) TFA only, (4) PITC to DMSO (1:1000 ratio PITC:DMSO) followed by TFA, and (5) same as (4) but with the additional trypsinization step.

### Amino acid read-outs: ClpS2-StV1 N-terminal binding and Glyphic antibody

#### Binding ClpS2-StV1 and anti hemagglutinin-tag (HA) antibody to different N-terminal peptides in gel

For Fig. 4a–d, ExM gels were cast at a concentration of 1mM peptide in the gelation solution, for all peptides: F_1_ peptide, W_1_ peptide, Y_1_ peptide, A_1_ peptide. This was done with 3 different aliquots of 0.8 mg lyophilized peptide (from AAPPTEC, > 95% purity) reconstituted to a concentration of 5 mM in water. The gels were made using a ~130 μm thick gelation chamber (i.e., 1 parafilm thickness). The gels were then expanded and re-embedded, as detailed in Methods: ExM gel: making the gelation mixture with peptide and Re-embedded ExM (ExMre) gels. The gels were cut to 2 × 2 mm top/flat surface size and stored at 4°C in 1X PBS, reaching a thickness of ~350 μm after expansion, with each gel containing ~70 pmol peptide for Fig. 4a–d, f–g. For **Supplementary Figure 7a**, the same peptides were reconstituted to a concentration of 25 mM in water to make 9% acrylamide gels using ~130 μm thick gelation chamber (i.e., 2 parafilm thickness) to reach a thickness of ~310 μm, cut to 2 × 2 mm with each gel containing ~4 nmol peptide. After 3 times 1X PBS washes, 10 min each, these gels were then placed in plastic microcentrifuge tubes (VWR, 87003-292) with 500 μL water and washed 3 times, 5 minutes each, with 500 μL water.

**Figure 1:**
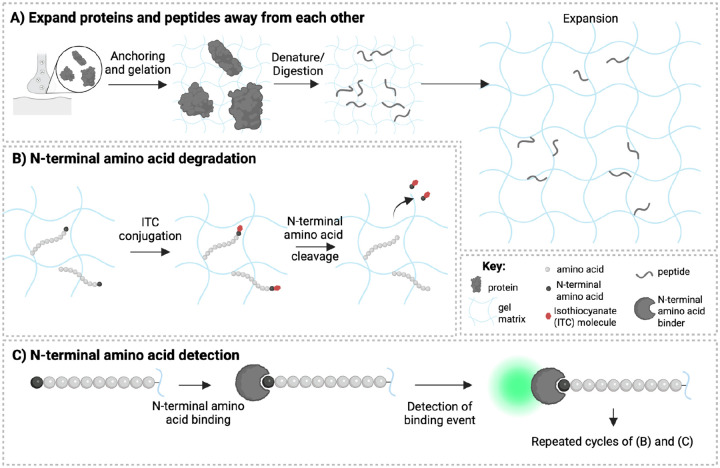
In-gel Edman degradation concept: a chemistry platform to support *in situ* protein sequencing technology development and application. (A) Expansion of the gel network to separate proteins and protein fragments, prior to peptide sequencing, exploits expansion microscopy (ExM)-style processing of a biological sample (e.g., a cell), including covalent anchoring key amino acids (e.g., lysine side chain modified with 6-((acryloyl)amino)hexanoic acid (AcX), in the original proExM protocol^6,7^), gelation, denaturation and/or digestion of proteins, and expansion, followed by re-embedding of ExM gels in charge-neutral gels to stabilize them in the expanded state (not shown). (B) Edman-in-gel chemistry can be conducted via conjugation of an isothiocyanate (ITC) derivative (e.g., phenylisothiocyanate (PITC)) to N-terminal amino acids of protein fragments or peptides, followed by cleavage of this N-terminal amino acid (e.g., with trifluoroacetic acid (TFA)). (C) The N-terminal amino acid at the end of a protein fragment or peptide can be detected with an N-terminal amino acid binder (e.g., ClpS2 St-V1^25,26^). The binding event is monitored (e.g., with antibodies with or without signal amplification, depicted as a green fluorescent signal next to the binder), before washing away of the binder and a new round of N-terminal amino acid degradation (B). Steps (B) and (C) are performed iteratively to sequentially identify the amino acids of the protein fragment or peptide.

**Figure 2:**
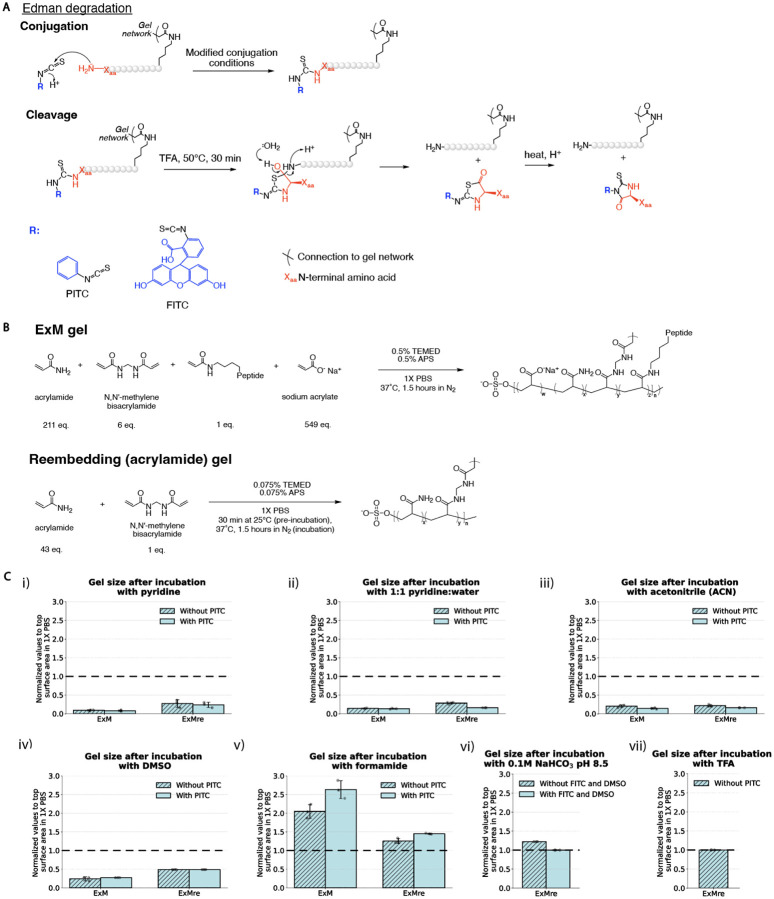
Edman degradation: chemical strategies for in-gel adaptation, and initial characterization of gel compatibility. (A) Proposed reaction scheme of Edman degradation within an ExM gel. To develop conditions, specific peptides were anchored to the gel. The sequencing chemistry includes conjugation of an isothiocyanate (ITC) molecule (i.e., Edman reagent) to the N-terminal amino acid (top). Edman degradation can be performed with a variety of isothiocyanates (denoted “R”, and depicted in blue). The conjugated N-terminal amino acid (denoted “Xaa”, and depicted in red) is then cleaved with TFA, eliminating the thiohydantoin amino acid derivative. (B) Reaction scheme for ExM, and re-embedding of ExM gels. Proteins will be anchored to the gel and then pulled apart from each other. We focus on using synthetic peptides with a C-terminal acryloyl functional group for covalent conjugation to the gel during free-radical polymerization. TEMED, tetramethylethylenediamine; APS, ammonium persulfate. (C) Flat/top side surface size of ExM and ExM re-embedded (ExMre) gels when placed in solvents used in Edman degradation: (i) 100% pyridine, (ii) 1:1 pyridine:water (all ratios throughout are of volumes added, unless otherwise indicated), (iii) acetonitrile (ACN), (iv) dimethylsulfoxide (DMSO), (v) formamide, (vi) 0.1 M sodium bicarbonate (NaHCO_3_). pH 8.5, (vii) trifluoroacetic acid (TFA), with and without Edman reagents (PITC 1:9 ratio PITC:solvent for (i), PITC 1:1000 PITC:solvent for (ii-v); FITC, 5.9 mM, and 23:77 DMSO:0.1 M sodium bicarbonate buffer pH 8.5, for (vi)). (Black dashed line: top/flat surface size of the gel in PBS; error bar: standard deviation; black dots, individual experiments; n=3 separate gelation solutions for (ii-vii); n=3 gels with same starting gelation solution for (i).)

A stock of ClpS2 St-V1 in a buffer of 50 mM sodium phosphate, 150 mM NaCl, pH 7.4 was made. The stock vial was spun down to remove any protein aggregates, and a concentration of ~116 μM was measured in the supernatant using NanoDrop (molecular weight of ClpS2 St-V1 is 13034 g/mol, and an extinction coefficient of 8940 M^−1^cm^−1^ at 280 nm). The protein sequence ClpS2 St-V1 (and for ClpS2, and ClpS2 V1) is in **Supplementary Note 6.2**. The protein was aliquoted and stored at −20 °C. Before use, the protein was diluted down to 10 μM in a buffer solution of 0.1% BSA in water, with ~4.3 mM sodium phosphate, 13 mM NaCl (for Fig. 4a, f–g), or 20 μM in a buffer solution of 0.1% BSA in water, with 8.6 mM sodium phosphate, 26 mM NaCl (for **Supplementary Figure 7a**). 30 μL of the solution was incubated with each gel for 6h (for Fig. 4a, f–g) or 1h (for **Supplementary Figure 7a**). For Fig. 4a,f–g, gels were washed with 0.1% BSA (Fisher Scientific, #NC1303417) in 1X PBS for ~1–3 min, and replaced with a 30 μL solution of 10 μg/mL (67 nM) (for Fig. 4a) or 20 μg/mL (133 nM) (for Fig. 4f–g) of anti-HA tag antibody 488 (ThermoFisher, Catalog # 26183-A488) incubated at 4 °C for 7 days (for Fig. 4a) or 3 days (for Fig. 4f–g). For Fig. 4a, after 5 days of antibody incubation, the gels were mixed with a pipette tip to make sure they were not stuck to one side of the plastic tube (which could disrupt equal diffusion of protein from either side of the gel). For Fig. 4f–g, after 1 day of antibody incubation, the gels were mixed with a pipette tip to make sure they were not stuck to one side of the plastic tube (which could disrupt equal diffusion of protein from either side of the gel). For **Supplementary Figure 7a**, gels were washed with 0.1% BSA in 1X PBS for ~1–3 min, and replaced with 100 μL solution of 200 μg/mL anti-HA tag antibody 647 (1.3 μM) (ThermoFisher, Catalog # 26183A647) incubated at 4 °C overnight (O/N).

#### Binding to HA-tagged peptides in acrylamide gels

For **Supplementary Figure 7b**, the HA-tagged peptides were reconstituted to a concentration of 5 mM in water to make 9% acrylamide gels using ~260 μm thick gel chambers (i.e., 2 parafilm thickness; as detailed in Methods: Acrylamide gel: making the gelation mixture with peptide). The gels were cut 2 × 2 mm top/flat surface size and stored at 4 °C in 1X PBS (reaching a thickness of ~310 μm in 1X PBS, with each gel containing ~750 pmol peptide). After 3 times 1X PBS washes, 10 min each, these gels were then placed in plastic microcentrifuge tubes with 500 μL water and washed 3 times, 5 minutes each, with 500 μL water. 30 μL of the 20 μM ClpS2St-V1 was incubated with each gel for 1h. The gels were washed with 0.1% BSA (Fisher Scientific, #NC1303417) in 1X PBS, and replaced with 30 μL solution of 20 μg/mL anti-HA tag antibody 647 (ThermoFisher, Catalog # 26183A647) incubated at 4 °C O/N.

#### In-gel Edman degradation for binding experiments with F_1_ peptide and G_1_F_2_ peptide ExMre gels

ExM gels were cast at a concentration of 1mM peptide, for both peptides: F_1_ peptide, and G_1_F_2_ peptide using 3 different aliquots of 0.8 mg lyophilized peptide (from AAPPTEC, > 95% purity) reconstituted to a concentration of 5 mM in water. The gels were made using 1 parafilm thickness gel chamber. The gels were expanded and re-embedded (as detailed in Methods: ExM gel: making the gelation mixture with peptide and Re-embedded ExM (ExMre) gels). ExMre gels were cut 5 × 5 mm squares and stored at 4 °C in 1X PBS. In-gel Edman degradation was performed on the gels as previously described (see section In-gel Edman degradation), except for the two following modifications: 1:9 of PITC:DMSO by adding 30 μL PITC to 270 μL DMSO, and with only 2 washes with DMSO, 5 min each, at 50 °C, between conjugation (PITC) and cleavage (TFA) steps. After TFA was removed, the gels were washed 3 times with 1M Tris pH 8, 10 min each, at RT. The gels for “PITC:DMSO+TFA+trypsin” condition were then submerged in 50 μL of 20 μg/mL trypsin in 50 mM Tris-HCl pH 8 in the bottom of the 1.5 mL Eppendorf tube (ensuring they were completely submerged in trypsin solution). The gels for other conditions were submerged in 50 μL of 50 mM Tris-HCl pH 8. All gels were then incubated for 2 hours at 37 °C. Each ExMre gel was further cut into 2 × 2 mm squares on a glass slide.

#### Glyphic binder specificity in gel and Biolayer Interferometry (BLI) curves

As depicted in Fig. 6d, antigens from Glyphic Biotechnologies were tested by embedding streptavidin in ExMre gels and subsequently adding biotinylated ClickP-amino acid (abbreviated: biotin-ClickP-aa). For AcX modification of streptavidin, 1.5 μL of 10 mg/mL AcX, (ThermoFisher, A20770) stored at −20 °C in DMSO, was added to 25 μL of 10 mg/mL streptavidin (ThermoFisher, 21122) resuspended in water and stored at 4 °C, to a total volume of 50 μL in 0.1 M NaHCO_3_ pH 8.5. This solution was left to incubate overnight at room temperature. Using the ~5 mg/mL AcX-modified streptavidin stock, ExM gels were cast, using ~130 μm thick gelation chamber (i.e., 1 parafilm thick), to a final concentration of ~0.1 mg/mL AcX-modified streptavidin. The gels were expanded and re-embedded (as in Methods: ExM gel: making the gelation mixture with peptide and Re-embedded ExM (ExMre) gels), to an approximate concentration of ~5 μg/mL (0.1 mg/mL / 2.7^3^) and stored in 1X PBS at 4 °C. The ExMre gels were cut into 2 × 2 mm squares, placed in separate plastic microcentrifuge tubes, and washed 3 times with water. The gels were incubated with biotin-ClickP-aa (where aa: G, V, F) at a concentration of ~16.7 μg/mL, freshly made from a stock of 0.5 mg/mL (from Glyphic) in a total volume of 100 μL in 1X PBS with 0.1% BSA for 24 hours at room temperature. These gels were washed 3 times, 20 min each, with 0.1% BSA in 1X PBS, and then incubated for 2 days with 30 μL of 10 μg/mL RFP-conjugated Glyphic antibody against ClickP-F (abbreviated: Glyphic F) or 5 μg/mL GFP-conjugated Glyphic antibody against ClickP-V (abbreviated: Glyphic V) in 0.1% BSA in 1X PBS at 4 °C.

BLI results of the Glyphic antibodies in **Supplementary Figure 9** were provided by the company.

#### Imaging

All imaging was performed using a spinning disk confocal microscope (Andor Dragonfly Spinning Disk confocal).

**Figure 3:**
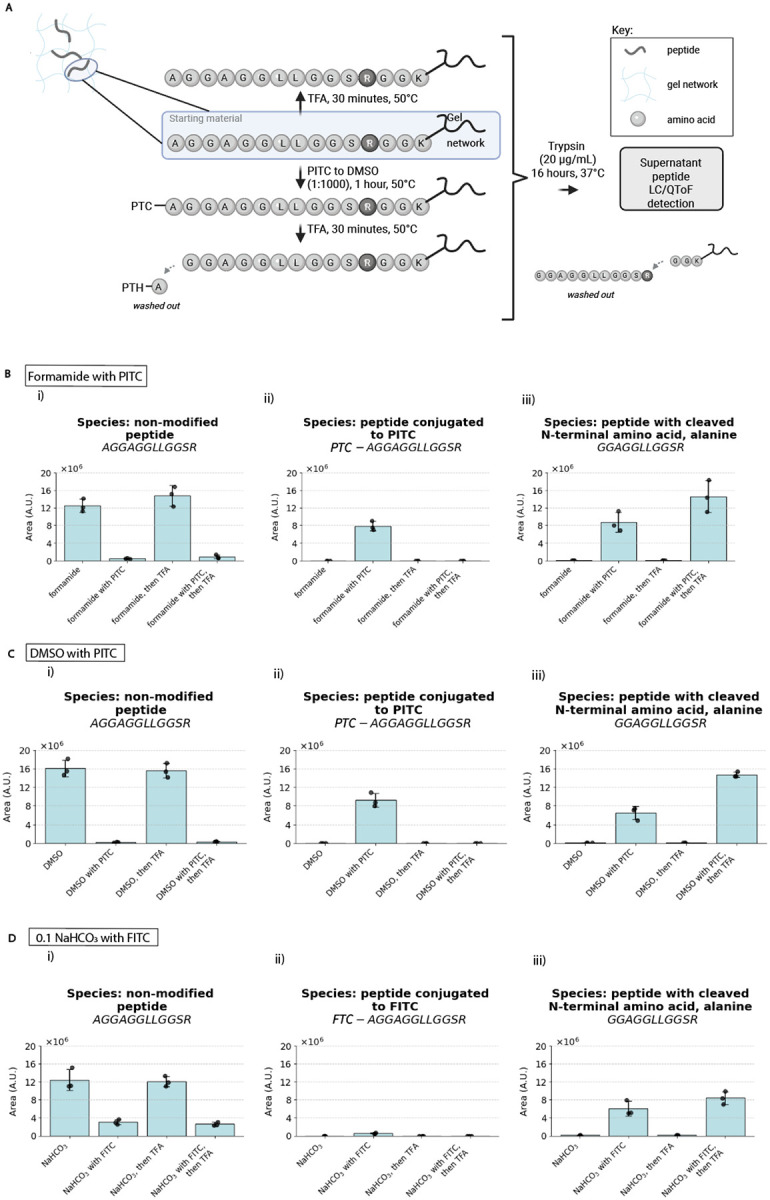

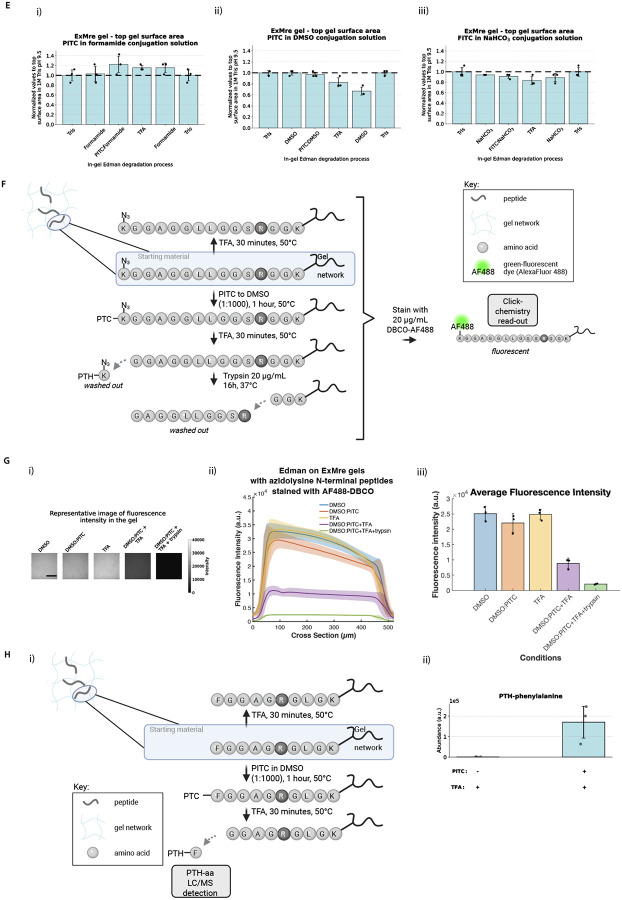
Assessing Edman degradation compatibility on peptides in ExMre gels using LC/QToF trypsinization assay, SPAAC fluorescence assay, and PTH-aa detection. (A) Assay to assess efficiency of Edman reagent conjugation on synthetic peptides in ExMre gels. Synthetic peptide AGGAGGLLGGSRGGK{acr} (abbreviated as A15-peptide; K{acr} denotes an acryloyl functional group on the lysine side chain); amino acids of the peptide chain are depicted as light grey beads, with arginine in the dark grey bead, as site of trypsin cleavage. Peptides are embedded into ExMre gels during free-radical polymerization of the swellable gel, and separate gels are subjected to various in-gel Edman conditions. All ExMre gels are then immersed in trypsin for digestion overnight to acquire the fragment upstream to the trypsin cleavage site at arginine, for further analysis. Supernatant is analyzed with liquid chromatography coupled with electrospray ionization quadrupole time-of-flight mass spectrometry (LC-ESI-QToF MS, abbreviated LC/QToF). (B) In-gel Edman chemistry with formamide as conjugation solvent and PITC as the Edman reagent. Bar graphs representing the relative abundance (arbitrary units, a.u., with all samples processed with spiked-in control compounds; see Methods and **Supplementary Figure 12**) of different peptide ion species detected on the LC/QToF. Bar graphs were obtained from measuring the area under the curve (AUC) of the chromatogram of various species (extracted based on the exact mass, see Methods for details, and **Source Data** for raw traces). The separate conditions were solvent only (“formamide”), PITC to solvent (1:1000 ratio PITC:solvent) for 1 hour at 50 °C (“formamide with PITC”), TFA for 30 min at 50 °C (“formamide, then TFA”), PITC to solvent (1:1000 ratio PITC:solvent) for 1 hour at 50 °C followed by TFA for 30 min at 50 °C (“formamide with PITC, then TFA”). The relative abundance of the ion species: (i) non-modified peptide (AGGAGGLLGGSR), (ii) peptide conjugated to PITC, phenylthiocarbamyl (PTC)-peptide (*PTC*-AGGAGGLLGGSR), and (iii) peptide with cleaved N-terminal amino acid (GGAGGLLGGSR), were reported throughout the in-gel Edman degradation process in the various conditions (dots, individual experiments; blue bar, mean; error bar, standard deviation, n=3 separate gelation solutions). (C) In-gel Edman chemistry as in B, but with DMSO as solvent. (D) In-gel Edman chemistry as in C, but with 23:77 of DMSO:0.1 M sodium bicarbonate pH 8.5 as conjugation solution with FITC (5.9 mM) as Edman reagent. The peptide conjugated to FITC is fluorescein-thiocarbamyl (FTC)-peptide (*FTC*-AGGAGGLLGGSR). (E) Top/flat surface size of ExMre gels throughout the Edman degradation process. Normalized top/flat surface size at each step for ExMre gels with (i) formamide conjugation with PITC, (ii) DMSO conjugation with PITC, or (iii) 5.9 mM FITC in 23:77 DMSO:0.1 M NaHCO_3_ pH 8.5 (thick dashed black line, top/flat surface size of the gel after washes in 1 M Tris pH 9.5 (1 mL × 3) before Edman degradation; error bar, standard deviation; black dots, individual experiments; n=3 separate gelation solutions). (F) Assay to assess efficiency of Edman degradation on synthetic peptides in ExMre gels. Synthetic peptide: K{N_3_}GGAGGLLGGSRGGK{acr} (abbreviated K{N_3_}15-peptide, where K{N_3_} is 6-azido-lysine), and amino acids of the peptide chain are depicted as light grey beads, with arginine, “R”, with the dark grey bead, as site of trypsin cleavage. The peptides are embedded into the ExMre gels during free-radical polymerization of the first gel, and separate gels are subjected to various Edman conditions. Read-out is performed using strain-promoted alkyne-azide cycloaddition (SPAAC) with dibenzocyclooctyne AlexaFluor 488 (DBCO-AF488) on the embedded peptides followed by analysis of the fluorescence. Note: sloped intensity profiles were likely due to excitation light attenuation in deeper layers of highly fluorescent gels. (G) ExM gels containing K{N_3_}15mer-peptide at 1 mM were cast in a gelation chamber, expanded and re-embedded to reach ~2.7X expansion factor at a final peptide concentration of ~50 μM. The separate conditions were DMSO only (“DMSO”), PITC to DMSO (1:1000 ratio PITC:DMSO; “PITC:DMSO”), DMSO followed by TFA, PITC to DMSO (1:1000 ratio PITC:DMSO) followed by TFA, and the same condition but with 20 ug/mL trypsin (“PITC:DMSO+TFA+trypsin”). After SPAAC with 20 μg/mL DBCO-AF488 in PBS, bulk gel fluorescence was imaged using a confocal microscope with a 10 μm Z-step. Analysis was performed on raw images. (i) A representative raw image of the fluorescence intensity is depicted for each gel condition, taking the 20th slice of the Z-stack for each (~200 μm deep into the gel). Scale bar is 500 μm. (ii) The fluorescence intensity of the ExMre gels in different conditions was compared throughout the gel thickness (~500 μm) (line, mean; shaded area, standard deviation; n=3 separate gelation solutions). (iii) Average fluorescence intensity of ExMre gels in the different conditions across the whole volume imaged (colored bar, mean; black dots, individual experiments; error bar, standard deviation, n=3 separate gelation solutions). (H) (i) Independent assay for in-gel Edman degradation, with phenylthiohydantoin (PTH)-F detection of Edman degraded synthetic peptide: FGGAGRGLGK{acr} (abbreviated “F_1_ peptide”) embedded in ExMre gels, as in (A). Separate gels were subjected to various Edman conditions. The conditions included TFA only for 30 min at 50 °C, and PITC to DMSO (1:1000 ratio PITC:DMSO) for 1 hour at 50 °C followed by TFA for 30 min at 50 °C. Subsequently, TFA was removed from the gels and they were immersed in 50 μL of 1:1 acetonitrile to water and agitated. Read-out was then performed by injecting the supernatant into LC/QToF using LC Method (see Methods). (ii) Results acquired as in (i). Analysis of PTH-F abundance was performed using PTH-F exact mass, 282.0827 ± 0.0056 Da (see **Methods for Edman degradation and PTH detection** for details). (blue bar, mean; error bar, standard deviation; black dots, individual experiments; n=3 separate gelation solutions).

For Fig. 3g, the gels were placed on a 24-well glass plate with 1 mL 1X PBS per well. Before imaging, the 1X PBS solution was removed, and the gel was imaged using a 10X magnification lens, acquiring a Z-stack covering the whole gel thickness, with a 10 μm Z-step size (520 μm total Z-stack, except condition “DMSO”, replicate 3, with a 510 μm total Z-stack), 40% laser power, 100 ms exposure time, with 488 laser line and 525/50 emission filter.

**Figure 4:**
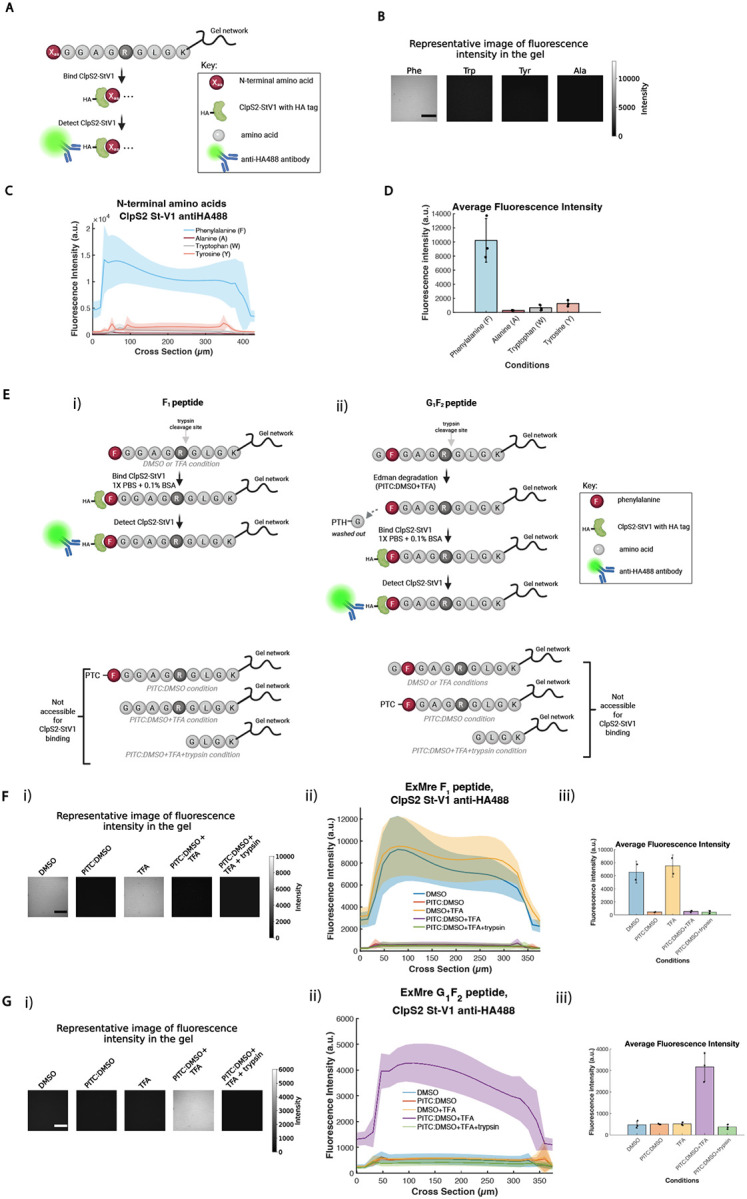
ClpS2 St-V1 serves as read-out for N-terminal phenylalanine on peptides in ExMre gels (A) Schematic representing different N-terminal peptides with otherwise similar sequence (**Xaa**GGAGRGLGK{acr}, where Xaa can be phenylalanine, alanine, tryptophan, tyrosine). When Xaa is F, abbreviated F_1_ peptide; A, abbreviated A_1_ peptide; W, abbreviated W_1_ peptide; Y, abbreviated Y_1_ peptide. The peptide is depicted as a chain of grey beads, with arginine “R” as the dark grey bead, and the N-terminal amino acid as a red bead. First, 10 μM ClpS2-StV1 protein with an HA tag is placed in solution to bind peptides in ExMre gels. The protein is briefly washed out, and the gels are stained with 67 nM anti-HA tag antibody conjugated to Alexa Fluor 488. Fluorescence of the gels then reports the relative binding of ClpS2-StV1 to the various N-terminal peptides via a population read-out using confocal imaging of the ExMre gels. (B) ExM gels containing F_1_ peptide, A_1_ peptide, W_1_ peptide and Y_1_ peptide are separately made at 1 mM concentration cast in a gelation chamber, expanded and re-embedded to reach ~2.7X expansion factor, reaching a final concentration of ~50 μM peptide (each gel with a volume of 1.4 μL). ExMre gels were incubated with 10 μM ClpS2-StV1, washed once, and placed in a solution of 67 nM anti-HA tag antibody 488 in 30 μL solution. The antibody solution is removed right before imaging on a confocal microscope, with a 10 μm step size covering the entirety of the gel. A representative raw image of the fluorescence of the gels is depicted for each ExMre gel containing a different N-terminal peptide, taking the 20th slice of the Z-stack for each (~200 μm deep into the gel). In black: scale bar, 100 μm. (C) Cross-section of the imaging performed in (B) for the different N-terminal peptides in ExMre gels after staining with ClpS2 St-V1 and anti-HA tag antibody 488. Raw intensity of the gels across the Z-stack were compared (line, mean; shaded area, standard deviation; n=3, with three different peptide aliquots, 3 different gelation mixtures). (D) Average fluorescence intensity of the images obtained in (B) for the different N-terminal peptides in ExMre gels after staining with ClpS2 St-V1 and anti-HA tag antibody 488. Fluorescence intensity of ExMre gels in the different conditions was averaged across the whole volume imaged (black dots, individual experiments; colored bar, mean; error bar, standard deviation, n=3, with three different peptide aliquots, 3 different gelation mixtures). (E) (i) F_1_ peptide, with the F amino acid depicted as a red bead, in ExMre gels. ClpS2-StV1 staining with anti-HA tag antibody 488 is performed as in (A). (ii) A peptide with sequence GFGAGRGLGK{acr}, abbreviated G_1_F_2_ peptide, where the F amino acid is depicted in red. 1 round of in-gel Edman degradation degrades N-terminal glycine (G) in ExMre gels. Then, ClpS2-StV1 and anti-HA tag 488 antibody staining is performed as in (A). (F) (i) A representative image of the fluorescence of the gels is depicted for each condition of in-gel Edman degradation for the F_1_ peptide, taking the 14th slice of the Z-stack for each (~210 μm deep into the gel, with 15 μm z steps). Imaging performed on a confocal microscope (~375 μm thick Z-stack). Scale bar, 100 μm. (ii) The gels in different conditions are compared in fluorescence intensity throughout their cross-section for the F_1_ peptide (line, mean; shaded area, standard deviation; n=2, different gelation solutions). (iii) Average fluorescence intensity of the ExMre gels in the different conditions for the F_1_ peptide by averaging across the whole volume imaged (black dots, individual experiments; colored bar, mean; error bar, standard deviation, n=2, different gelation solutions). (G) (i) As in Fi, but with G_1_F_2_ peptide, taking the 13th slice of the Z-stack for each (~195 μm deep into the gel, with 15 μm z steps). (ii) As in Fii, but with G_1_F_2_ peptide (line, mean; shaded area, standard deviation; n=3, different gelation solutions). (iii) As in Fiii, but with G_1_F_2_ peptide (black dots, individual experiments; colored bar, mean; error bar, standard deviation, n=3, different gelation solutions).

**Figure 5:**
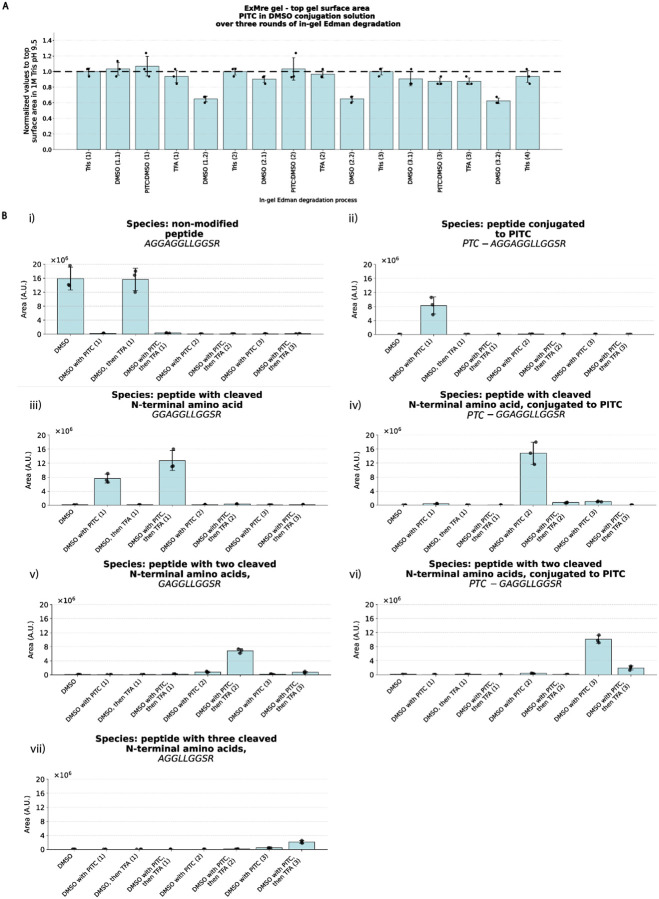
In-gel Edman degradation over multiple rounds using LC/QToF trypsinization assay (A) Top/flat surface size of ExM and ExMre gels when placed in solvents used in Edman degradation. Normalized top/flat surface size to top/flat surface size in 1X PBS throughout 3 rounds of in-gel Edman degradation for ExMre gels with 1:1000 ratio PITC to DMSO. The round number is specified in parentheses (eg., “1”), and if solution is used at various steps of the same round, the first and second submersion of the gel is specified with an additional number (eg., “1.2”). (Thick dashed black line: top/flat surface size of the gel in 1M Tris pH 9.5 before Edman degradation, error bar: standard deviation, black dots, individual experiments, n=3, three separate gelation solutions.) (B) Bar graphs representing the relative abundance (arbitrary units, a.u.) of different peptide ion species from the LC/QToF, obtained from the area under the curve (AUC) of the chromatogram based on the exact mass of the various species (Agilent MassHunter Qualitative Analysis software). The relative abundance of the ion species: (i) non-modified peptide (AGGAGGLLGGSR), (ii) peptide conjugated to PITC (*PTC*-AGGAGGLLGGSR), (iii) peptide with cleaved N-terminal amino acid (GGAGGLLGGSR), (iv) peptide with cleaved N-terminal amino acid conjugated to PITC (*PTC*-GGAGGLLGGSR), (v) peptide with two cleaved N-terminal amino acids (GAGGLLGGSR), (vi) peptide with two cleaved N-terminal amino acids conjugated to PITC (*PTC*-AGGLLGGSR), (vii) peptide with three cleaved N-terminal amino acids (AGGLLGGSR), are reported throughout the Edman degradation process in the gel in the various conditions. Conditions include: DMSO only, DMSO with PITC, TFA only, and sequential DMSO with PITC followed by TFA (black dots, individual experiments; blue bar, mean; error bar, standard deviation, n=3, three separate gelation solutions).

For Fig. 4b–d and Fig. 4f–g, the supernatant antibody solution was first removed from the gels via a transfer pipette before imaging. They were placed on a droplet of 50 μL of 1X PBS with 0.1% BSA on a 24-well plate for the smooth transfer of the gel, and the droplet was then removed after the transfer. Imaging was performed with 488 laser and an emission filter of 525/50, with 100% laser power and 500 ms exposure time, using a 40X objective (water immersion) and 10 μm step size (Fig. 4b–d) or 15 μm step size (Fig. 4f–g) covering the entirety of the gel cross-section (for a final ~430 μm Z-stack for Fig. 4b–d; **~**375 μm Z-stack for Fig. 4f–g).

**Figure 6:**
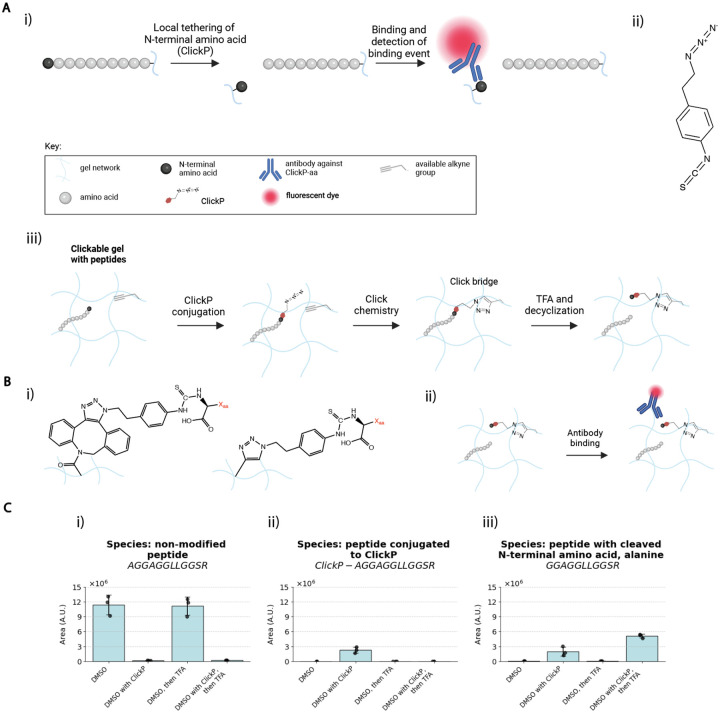

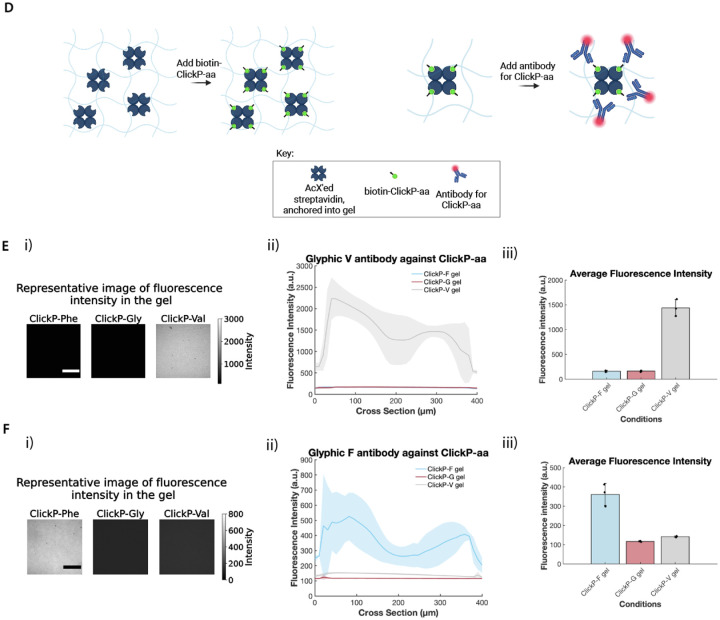
ClickP as Edman reagent, and ClickP-specific binders in ExMre gels. (A) (i) 1-(2-azidoethyl)-4-isothiocyanatobenzene, abbreviated ClickP ITC derivative, is used for local tethering of the ClickP-conjugated N-terminal amino acid to the polymer network, followed by cleavage with TFA. Binders (eg., commercially available Glyphic antibodies) are used to detect the cleaved N-terminal amino acid bound to the polymer network. N-terminal amino acid local tethering followed by detection are performed iteratively to sequentially identify the amino acids of the chain. (ii) The structure of the ClickP ITC derivative. (iii) Peptides cast in an ExMre hydrogel conjugated with “clickable” groups (denoted: “Clickable gel”, e.g. with alkyne groups for copper-catalyzed chemistry). The bifunctional molecule, with an azide and isothiocyanate group, first reacts to primary amines at the N-terminus of peptides via the isothiocyanate group. Then, the copper-catalyzed click chemistry forms a bridge between the ClickP-conjugated N-terminus (4-(2-azidoethyl)phenylthiocarbamoyl-peptide) via the azide group with the alkyne group in the gel. TFA is then used to cleave the N-terminal amino acid from the peptide, and the covalent click reaction locally tethering the amino acid to the surrounding hydrogel network, prevents its diffusion out of the gel. The N-terminal amino acid is decyclized from the thiohydantoin or thiozolinone intermediates, from post-cleavage, into the thiocarbamoyl derivative with covalently tethered N-terminus and an available C-terminus (carboxyl group). (B) (i) The final antigen the Glyphic antibodies recognize. Either a reacted DBCO-azide click or a reacted alkyne-azide click group, with a given amino acid and its side chain exposed (amino acid side chain: “Xaa”, in red text), including the free C-terminus of the amino acid. (ii) Antibody binding can be performed after the N-terminal amino acid is tethered locally to the surrounding hydrogel network, cleaved from the peptide, and decyclized into the PTC form. (C) Bar graphs representing the relative abundance (arbitrary units, a.u.) of different peptide ion species from the LC/QToF, obtained from the area under the curve (AUC) of the chromatogram based on the exact mass of the various species (Agilent MassHunter Qualitative Analysis software). The relative abundance of the ion species: (i) non-modified peptide (AGGAGGLLGGSR), (ii) peptide conjugated to ClickP (4-(2-azidoethyl)phenylthiocarbamoyl-peptide, abbreviated: *ClickP*-AGGAGGLLGGSR), and (iii) peptide with cleaved N-terminal amino acid (GGAGGLLGGSR), is reported throughout the Edman degradation process in the gel in the various conditions (black dots, individual experiments; blue bar, mean; error bar, standard deviation, n=3, three separate gelation solutions). (D) Schematic of the binding assay with biotin-ClickP-aa in ExMre gels with streptavidin. ExMre gels are cast with AcX-reacted streptavidin protein. Separate gels are made for each tested amino acid, by adding biotin-ClickP-amino acid (abbreviated: biotin-ClickP-aa), where the aa is either phenylalanine (Phe, or F), glycine (Gly or G), or valine (Val or V), to bind the streptavidin protein embedded in the gel (left). After the binding of biotin-ClickP-aa to the gels, which exposes the final antigen that Glyphic antibodies recognize, the GFP-conjugated Glyphic antibody against ClickP-V (abbreviated: Glyphic V) and RFP-conjugated Glyphic antibody against ClickP-F (abbreviated: Glyphic F), are added to the gels to assess specificity of binding towards the various ClickP-aa (right). (E) Binding assay with biotin-ClickP-aa (aa: G, F, V) in ExMre gels with streptavidin using Glyphic V. First, streptavidin is embedded into ExM gels at a concentration of 0.1 mg/mL and after expansion and re-embedding reach a concentration of ~5 μg/mL. Biotin-ClickP-aa is added at a concentration of ~17 μg/mL. Then, Glyphic V is added at concentration of 5 μg/mL. (i) A representative raw image of the fluorescence of the gels is depicted for the gels in different conditions, comparing binding of Glyphic V against biotin-ClickP-F, biotin-ClickP-V and biotin-ClickP-G ExMre gels obtained from a confocal microscope, showing a slice 90 μm deep into the gel. Scale bar in white for the ClickP-Phe image (left): 100 μm. (ii) The ExMre gels in different conditions are compared in fluorescence intensity throughout the Z-stack (line, mean; shaded area, standard deviation; n=3 different gelation solutions). (iii) Average fluorescence intensity of ExMre gels in the different conditions by averaging across the whole volume imaged (black dots, individual experiments; colored bar, mean; error bar, standard deviation, n=3 different gelation solutions). (F) As in E, but using Glyphic F (black dots, individual experiments; colored bar, mean; error bar, standard deviation, n=3 different gelation solutions).

For Fig. 6e–f, the gels were placed on a 24-well plate on a droplet of 50 μL 1X PBS 0.1% BSA in the center of the well to transfer the gel before imaging. Imaging was performed with a 40X objective (water immersion), and a Z-stack obtained from 10 μm step size across the depth of the gels (or a final ~400 μm Z-stack) using 500 ms exposure time, 100% laser power with excitation wavelength in 488 channel and an emission filter of 525/50 (Glyphic V antibody) or 561 and an emission filter of 582/15 (Glyphic F antibody).

For **Supplementary Figure 7a**, the imaging was performed after removing the antibody (anti-HA tag antibody 647) solution using a confocal microscope with 10X objective, 100% laser power in 637 laser with 676/37 emission filter, 400 ms exposure time, 10 μm Z-steps covering the entire gel cross-section.

For **Supplementary Figure 7b**, the imaging was performed after removing the antibody (anti-HA tag antibody 647) solution using a confocal microscope with 10X objective, 100% laser power in 637 laser with 676/37 emission filter, 100 ms exposure time, 10 μm Z-steps covering the entire gel cross-section.

#### Image analysis

For Fig. 3g, Fig. 4b–d, Fig. 4f–g and Figure 6e–f, images (‘.ims’ files) were opened in FIJI and gel Z-stacks were inspected by plotting the Z-axis profile. The final Z-stack thickness for all conditions was set to capture the entire thickness of the gel and was made identical for each replicate (see Imaging section). The images were then saved as ‘.tif’ files.

The plots of gel fluorescence throughout the Z-stack (Fig. 3gii, Fig. 4c, fii, gii, and Fig. 6eii, fii) and bar plots of the average fluorescence throughout the gel volume (Fig. 3giii, Fig. 4d, fiii, giii, and Fig. 6eiii, fiii) were made in MATLAB (see GitHub **Code**). For the Z-stack plot showing fluorescence throughout the gel, the mean and standard deviation of fluorescence for each Z-slice (over the replicates of the same condition for the same Z-slice) was calculated and plotted in different colors for each condition. For the bar plots, the average fluorescence of the gels across the whole volume was computed (i.e., for each Z-slice of a given sample, the mean fluorescence intensity of all pixel values was computed. Then, for each sample, the average intensity across all its Z-slices was calculated, and plotted as a separate data point. Finally, the average intensity for each condition was obtained by computing the mean of these sample-wise average intensities).

The representative images of each gel (Fig. 3gi, 4b, 4fi, 4gi, 6ei, 6fi) was obtained from one gel replicate for a given condition. The same arbitrarily selected depth, reported in the figure captions, was taken for each condition by making a substack of a single Z-slice (e.g. to obtain the image for Z-slice # 20: Image → Stacks → Tools → Make Substack, input “20–20”). A scale bar was also added to every image representing the first condition of each experiment. These average intensity profiles for each condition were then plotted in Python using the *mpimg* Matplotlib image module, with an intensity scale for comparison (see GitHub **Code**).

#### Oxidation analysis in ExM gels

Amino acid oxidation analysis in ExM gels were performed with XaaGGAGRGLGK{acr} peptides, where Xaa was one of 8 amino acids: M, C, W, Y, F, P, H, R (as described in Synthetic peptide designs section). Post-translational oxidation modifications for each amino acid can be found in **Supplementary Figures 8a-h**.

Three conditions were prepared for each of the N-terminal acid peptides. Two in solution, where condition 1 was: 20 μL of 5 mM peptide with 90 μL water, and condition 2 was: 20 μL of 5 mM peptide with 80 μL water, 5 μL of 10% TEMED (v/v), and 5 μL 10% APS (w/v), incubated at 37 °C for 1h30m. The condition 3 was ExM gelation: 20 μL of 5 mM peptide with 60 μL of StockX, 10 μL of 130 mM BIS, 5 μL of 10% TEMED (v/v), and 5uL 10% APS (w/v) gelled into 20 × 10 mm surface size chamber with ~260 μm chamber thickness (i.e., 2 parafilm thickness). Gelation was performed as described in section ExM gel: making the gelation mixture with peptide.

Condition 1 and condition 2 were further diluted by taking 15 μL of the sample and adding 50 μL 20 μg/mL trypsin in 1M Tris pH 8 for a total volume of 65 μL. Condition 3 gels were cut into 5 mm × 10 mm ExM gel using a razor blade (right after gelation; without expanding) and washed twice with 1X PBS, 5 min each, then once with 1M Tris pH 8 for 5 min. The wash solution was then replaced with 50 μL of 20 μg/mL trypsin in 1M Tris pH 8 on top of the gel. Condition 1, condition 2 and condition 3 were all incubated for 4 hours at 37 °C with the trypsin solution. After trypsinization, the samples were placed in the fridge until analysis on LC/QToF. 50 μL of the solutions, for each condition, were placed in separate mass spec vials for analysis. Condition 1, 2, and 3 samples were injected into the LC/QToF (see section on Mass spectrometry).

Analysis of the N-terminal amino acid oxidation was performed as detailed in **Analysis of LC/QToF data** using the information about post-translational oxidation modification of peptides documented in **Supplementary Table 3**. For each N-terminal amino acid, the abundance (a.u.) of the original fragment and the fragment with various post-translational oxidation modifications was extracted for the three different sample conditions and documented in a table (see **Supplementary Figures 8aii-hii**). Then, using these values, the relative abundance of each of the fragments were compared using a bar graph. For this, the abundance of each species was normalized with max absolute scaling for plotting (see **Supplementary Figures 8aiii-hiii**). The raw total ion chromatograms and mass spectra were exported as metafiles (‘.emf’), and available in **Source Data**.

### Theoretical assessment of *in situ* protein sequencing

All analyses were performed either on mycoplasma genitalium proteome (483 Swiss-Prot reviewed proteins; UniProt ID 243273: by searching “taxonomy_id:243273” in UniProt), or the human proteome (20,421 Swiss-Prot reviewed proteins, UniProt ID 9606: by searching “taxonomy_id:9606” in UniProt). All references throughout for mycoplasma proteome and the human proteome are for these two proteomes, respectively. All code can be found on GitHub, at: https://github.com/camimarie/insituprotein/. Amino acid residues are abbreviated using their single letter amino acid code. N-terminal is abbreviated “N-term”.

### Part 1: results of exploring the chemistries and their effects on preserving protein sequence coverage in the gel

#### Percent amino acid count

For **Supplementary Figure 13c** (mycoplasma proteome) heatmap, the protein database went through a three step chemical process that modified the primary sequence of amino acids. A gridsearch was performed with various combinations of P(fix), P(anchor), P(digest), where these represent the probability of these three chemical steps to modify a subset of amino acid side chains. In addition, the anchoring could be either AcX or epoxide, and digestion could be either endoprotease Lys-C (abbreviated Lys-C), Proteinase K (abbreviated ProK) or trypsin. The gridsearch values were: P(fixed): 0.00, 0.01, 0.05, 0.10, 0.50, 1.00; P(anchored): 0.0, 0.1, 0.2, 0.3, 0.4, 0.5, 0.6, 0.7, 0.8, 0.9, 1.0; P(digestion): 0.0, 0.1, 0.2, 0.3, 0.4, 0.5, 0.6, 0.7, 0.8, 0.9, 1.0. First, the proteome (mycoplasma or human) was selected from UniProt and loaded into the computational environment. Then, N-term, K, C, R, and Y residues were modified with probability P(fixed), where they were replaced with a “J” (arbitrarily, since “J” is not overlapping with any other amino acid single letter code) after fixation. Similarly, for anchoring, N-term and K residues for AcX, or N-term, K, C, H, Y, D and E for epoxide, were modified with probability of P(anchored), and replaced with a “Z” (since “Z” was not overlapping with any other letter). Lysine residues that were already converted to a J could not be modified to a Z in this anchoring step). For digestion, residue K was digested at its C-terminus for Lys-C, residues R and K were digested at their C-terminus for trypsin, whereas for ProK, Y, W, E, L, V, A, I, F, T residues were digested at their C-terminus. For all digestion types (Lys-C, trypsin, ProK), digestion did not occur if they had already been converted to a J (fixed) or Z (anchored). A fragment was only retained if it had at least one anchored residue to the gel matrix from the P(anchor), otherwise it was considered lost. Each grid in the grid search represents a unique combination of those three parameters, where each unique combination was run 10 times. The “percent amino acid count” was calculated as the percentage of residues of a protein that are not modified (i.e., remains a canonical amino acid), and remain accessible for read-out over 15 rounds of in-gel Edman degradation after the chemical steps. This was computed by summing over all the amino acids of a fragment that are unmodified by any chemical process, stopping at the last anchor of a fragment (subsequent amino acids would be lost and not be readable by in-gel Edman degradation) and, if the final anchor is not within the first 15 amino acids of the fragment, stopping at the 15th amino acid. The N-term modifications in either the fixation or anchoring steps prevented read-out of the given fragment, for the mycoplasma proteome. The mean percent amino acid count was calculated by averaging over the 10 replicated computational experiments and also over the proteome and plotted on the heatmap in **Supplementary Figure 13c.**

For **Supplementary Figure 18** (human proteome), it was the same as described for **Supplementary Figure 13c** (mycoplasma proteome), but the N-terminal fragment was always considered inaccessible due to N-terminal acetylation. In addition the gridsearch values for P(fixed), P(anchored) and P(digestion) were the same values as above.

For **Supplementary Figure 14a** and **Supplementary Figure 14c**, the distribution of the percent amino acid count (defined above) was plotted as a distribution for all the proteins in the proteomes averaged over the 10 rounds of computational experiments for P(fixation)=0.05, P(anchoring)=0.80, P(digestion)=0.80, for both mycoplasma and human proteomes, where the error bar represented the standard deviation over the multiple computational repetitions. For **Supplementary Figure 14b, Supplementary Figure 14d**, the distribution of the percent amino acid count was plotted as a distribution for all the proteins in the proteomes over the 10 rounds of computational experiments for the highest percent amino acid count for each condition (best performing parameters given the condition) as calculated and plotted in **Supplementary Figure 13c** and **Supplementary Figure 18** (for the mycoplasma proteome and human proteome, respectively). The bin-size is 40.

#### Fragment lengths

For **Supplementary Figure 15**, the fragment length was defined as the number of amino acids for a given fragment retained in the gel, including amino acids that are fixed or anchored (unlike the percent amino acid count), but not considering amino acids that are located downstream, C-terminal, of the last anchoring amino acid of the fragment. We also do not cap at 15 amino acids, unlike the percent amino acid count. The results for these fragment lengths were plotted as distributions of protein count versus fragment length, where the mean and median values were recorded by plotting a vertical line along with their values. The y-axis was log-scale, and standard deviations were the result of 10 computational repetitions, with bin-size of 40. Results of the fragment lengths using a parametrization of P(fixation)=0.05, P(anchoring)=0.8, P(digestion)=0.8, were plotted for the mycoplasma proteome, human proteome (**Supplementary Figure 15a-b**, respectively), which were the same parameterizations used in **Part 2**.

### Part 2: Simulating *in situ* protein sequencing with NAABs and in gel Edman degradation

#### Reference fragment dataset ${(T}_{s})$

The reference fragment dataset represents the database of ground-truth sequences present in the gel, after fixation, anchoring, gelation, digestion and expansion, but prior to the *in situ* protein sequencing read-out. To generate the dataset, first, similarly to **Part 1** methods, the mycoplasma proteome was selected from UniProt and loaded into the computational environment. Then, the primary sequence of amino acids was modified according to parameters for P(fixed), P(anchored), P(digestion), where these represent the probability of these three chemical steps to modify a subset of amino acid side chains. However, in **Part 2**, these values were fixed with P(fixed)=0.05, P(anchored)=0.8, P(digestion)=0.8 using paraformaldehyde, AcX and trypsin for fixation, anchoring and digestion in the model, respectively. The amino acid side chain specificities of the paraformaldehyde fixation, anchoring with AcX, and digestion with trypsin, remained the same as in **Part 1**. Only fragments with at least one anchored residue to the gel matrix from the P(anchoring) were retained. Subsequently, an idealistic in-gel Edman degradation read-out was conducted on the remaining fragments by considering all the sequencing chemistries, including the conjugation and cleavage of in-gel Edman degradation to be 100%. In addition, the idealistic read-out could detect all amino acids with 100% accuracy, except ones that have been fixed or anchored at previous steps (fixation and anchoring). In this way, the fragments generated were perfectly corresponding to the ground-truth sequence that would be expected when reading them out in idealistic conditions. In addition, if Edman leads to the loss of the only anchor, amino acids following are gone and will be substituted with an unknown read (“X”) since there will be no further amino acids to readout. After, amino acid sidechains modified to J or Z are unrecognized by binders, and thus will return X (given that modifications like J and Z cannot be distinguished during read-out, and will also result in failure to bind) and stored as a fragment. This was performed 1,000 times, and the fragments from these computational experiments were then aggregated into the reference fragment dataset, also named $T_{s}$ (peptides anchored in the expansion gel independent of read errors). For **Supplementary Figure 16**, the number of unique fragments generated were compared for 1, 10, 100, 1,000, and 10,000 simulations and plotted for mycoplasma.

#### Error-prone fragment dataset and assignment to proteins

The proteome (mycoplasma) was selected from UniProt and loaded into the computational environment. The first steps of the error-prone fragment dataset were the same as the reference fragment dataset generation up until the modification of the primary protein sequences into fragments through digestion (with values for P(fixed)=0.05, P(anchored)=0.8, P(digestion)=0.8 using paraformaldehyde, AcX and trypsin for fixation, anchoring and digestion in the model, respectively). Only fragments with at least one anchored residue to the gel matrix from the P(anchoring) were retained. After, amino acid sidechains modified to J or Z are unrecognized by binders, and thus will return X (given that modifications like J and Z cannot be distinguished during read-out). At this stage, instead of considering an idealistic in-gel Edman degradation read-out, the read-out was simulated with errors, including the sequencing chemistry and the binder kinetics.

#### Sequencing chemistry errors

Specifically, for the sequencing chemistry errors, the conjugation of PITC was considered to have a probability of success of 0.9, and the cleavage with TFA with a 0.7 success rate. Amino acids with a modified sidechain (by anchoring or fixation) were considered to be cleavable via in-gel Edman degradation, but when reaching the last anchor of the peptide, the rest of the fragment was lost and subsequent amino acids would be read as unknown (i.e. “X”).

#### Binder read-out errors and error-prone fragment dataset ${(F}_{s})$

For simulation of the binding, after the PITC conjugation step, binders were bound probabilistically to PITC-conjugated N-terminal amino acids, and this was performed sequentially for every binder. Results were performed with several subsets of binders that recognized various PITC-conjugated N-terminal amino acids. The 20 amino acid binder subset with recognizers towards all canonical amino acid side-chains (but not the X sidechains, of course). The 15 amino acid binder subset contained the following recognizers: {A, N, D, E, Q, G, I, L, K, F, S, T, P, Y, V}, the 10 amino acid binder subset contained the following recognizers: {A, N, D, E, Q, I, L, K, F, V}, and the 5 amino acid binder subset contained the following recognizers: {N, E, L, F, V}. The binding was simulated in a condition with excess binder, and where binding reaches equilibrium (using the Langmuir equation for binding), such that after the washing, the probability of being bound to on-target and off-target was defined as: $p_{\text{correct}}=\left(\frac{\left[C\right]}{[C]+{K_{d}}^{\text{on}-\text{target}}}\right)\cdot e^{-{k_{off}}^{on-\text{target}}t_{\text{wash}}}$ and $p_{\text{incorrect}}=\left(\frac{\left[C\right]}{[C]+{K_{d}}^{\text{off}-\text{target}}}\right)\cdot e^{-{k_{off}}^{off-\text{target}}t_{\text{wash}}}$, where [C] is the concentration of binder, ${K_{d}}^{\text{on}-\text{target}}$ the equilibrium dissociation constant for the on-target, ${K_{d}}^{\text{off}-\text{target}}$ the equilibrium dissociation constant for the off-target, ${k_{off}}^{on-\text{target}}$ the dissociation rate for the on-target, and ${k_{\text{off}}}^{\text{off}-\text{target}}$ the dissociation rate for the off-target, and $t_{\text{wash}}$ is the time elapsed during the dissociation phase (during the washing). **Supplementary Figure 17** was plotted with these equations (plotting $p_{\text{correct}}-p_{\text{incorrect}}$) by modifying the values of binder washing, $t_{\text{wash}}$ from 0 to 200 min, and the concentration of binder, [C], from 1 pM to 100 mM. These were plotted for a binder with different levels of specificity according to table **Supplementary Table 12**: very high, high, medium, low, and very low specificities, where the equilibrium dissociation constant and dissociation rates for on-target and off-target were modified in the equation for each of these specificity levels. Very high, high, and medium resulted in the same on-target and off-target binding profiles, and thus only one (medium) specificity was included in **Supplementary Figure 13d**. For the results in **Supplementary Figure 13d** (mycoplasma proteome), the $t_{\text{wash}}$ was considered as 30 min, and the [C] as 1 μM, and results were generated for different levels of binder specificities according to table **Supplementary Table 12**. For each in-gel Edman degradation round, independent reads were obtained for each amino acid (20, 15, 10, and 5 times) since the binding was performed sequentially for each binder. Thus, several read-outs were associated with the same position in a peptide fragment (this was taken into consideration for the downstream Hidden Markov Model (HMM)). After performing the binding, the N-terminal amino acid was cleaved with sequencing chemistry errors mentioned above, and the process was repeated from 5 to 15 rounds of in-gel Edman degradation and sequential binder read-out. This resulted in error-prone fragment sequences for each protein, which collectively became the error-prone fragment dataset ${(F}_{s})$.

### Hidden Markov Model (HMM) based matching and fraction of proteome correctly identified

#### Pre-filtering uninformative fragments

Following the generation of the error-prone dataset, which included for every protein in the mycoplasma proteome at each condition (number of rounds × number of binder cases × number of specificity cases × 10 repetitions = 1,760 conditions), low quality fragments were filtered. First, uninformative “query fragments”, originating from the error-prone dataset $F_{s}$, were defined as fragments that consisted of more than half of their total Edman degradation rounds contained only unknown reads (“X”), e.g. rounds where no binder sequentially bound at all. These fragments were removed. Second, uninformative “reference fragments”, originating from the reference dataset $T_{s}$, were defined as fragments that consisted of more than half of the total reads as unknown reads, denoted by an “X”. These fragments were also removed.

#### Algorithmic details for two-stage HMM-based matching:

Next, for each remaining fragment, a two-stage HMM-based matching was performed. The HMM models the joint probability P(F∣T,θ) that a true fragment T generates an observed fragment F given the experimental error parameters θ, which include the following:

- Conjugation failures (c): probability that no conjugation occurs in an Edman round.
- Cleavage failures (d): probability that the N-terminal amino acid is not cleaved.
- Binder errors: per-binder probabilities describing on and off-target binding, where unbound is denoted as “X”: $p_{\text{bound},\text{on}-\text{target}},p_{\text{unbound},\text{on}-\text{target}},p_{\text{bound},\text{off}-\text{target}},p_{\text{unbound},\text{off}-\text{target}}.$

At each Edman round, each amino acid from the true fragment T emits m observations, one per binder in the binder sets described above. The model sums over all possible emission paths and state transitions, including insertion (failed cleavage) and stay (failed conjugation) transitions.

To map each observed fragment, subject to Edman conjugation failures, Edman cleavage failures, and binder errors, back to its source, we framed the problem as follows: for every “true” fragment T in the error-free trie, $T_{s}$ (reference fragment dataset), what was the probability that T under set parameters θ would generate the observed, error-prone fragment F in the error-prone dataset $\left(F_{s}\right)$. Thus, for each error-prone fragment T, the Viterbi log-likelihood, $\underset{\pi }{\text{max}}\;\text{log}\;P(F,\pi |T,\theta )$ was computed, which represents the probability of the most likely path π* generating the observed fragment F, given each candidate sequence T. After computing these Viterbi scores for all candidates, we apply a pruning step to discard less likely candidates: $\text{log}\;P\left(F,\pi *|T,\theta \right)\geq \underset{S^{\prime }}{\text{max}}\;\text{log}\;P\left(F,\pi *|S^{\prime },\theta \right)-\epsilon_{vit}$, where $S^{\prime }$ is the highest scoring fragment across all candidates. $\epsilon_{\text{vit}}$ was set as 5.0, which was chosen to be deliberately wide as there is increased variance with certain conditions: in log-likelihood space, any candidate that scores $1/e^{5}\approx 1/150$ of the top score is retained. On the pruned candidate set, a forward dynamic programming algorithm is employed that computes the total likelihood $\text{log}\;L(T\to F)=\text{log}\;P(F|T,\theta )=\text{log}\;\sum_{\pi }P(F,\pi |T,\theta )$, summing over almost all possible paths with a milder beam $\epsilon_{\text{for}}=10.0$. Note that no candidates are ever removed here, as the forward epsilon only prunes unlikely paths within the possible paths.

#### Fraction of the proteome correctly identified

For each of the top 10 candidate fragments by forward log-likelihood $T\in T_{S}$ (if such fragments exist and have not been filtered out), we log-summed fragment likelihoods weighted by counts using the reference fragment dataset’s protein occurrence counts for each fragment T. We then normalize and rank proteins by their total weight. The highest scoring protein is returned as the fragment’s originating protein only if the weight is at least 1.5x the second-highest scoring protein. Otherwise, the fragment is labeled “uncertain”. For each protein, we accumulate all the fragments’ labels. If no valid predictions are produced, we return “no predictions”. Otherwise, we count fragment-level votes per predicted protein: the protein is identified only if the top vote count is at least double the runner up, otherwise we return “uncertain”. We report the counts of “correct”, “false positive”, “uncertain”, and “no predictions”. From these decisions, the fraction correctly identified over the whole proteome was a metric for how many of the proteins in the mycoplasma proteome were correctly identified from the simulation of the error-prone fragments (not including uncertain or false positive matches). For **Supplementary Figure 13d**, the whole simulation was performed 10 times and the average and standard deviation of the fraction of correctly identified proteins was reported on the graph of results. In **Supplementary Figure 19**, we plot the average and standard deviation of the fraction of false positively identified proteins.

## Figures and Tables

**Table 1:** Acrylamide gelation solution

Acrylamide gelation solution	Concentration	Final concentration	Add
Acrylamide	50% (w/v)	9% (w/v)	20 μL
BIS	130 mM	9.1 mM	7.7 μL
PBS	10x	0.9x	10 μL
Water			62.3 μL
APS	10% (w/v)	0.45% (w/v)	5 μL
TEMED	10% (v/v)	0.45% (v/v)	5 μL
Total volume			110 μL

**Table 2:** StockX monomer solution

StockX monomer solution	Concentration	Amount (mL)
Sodium acrylate (Combi-Blocks QC-1489)	38% w/v	2.25
Acrylamide	50% w/v	0.5
BIS	2% w/v	0.75
Sodium chloride (J60434.AK)	5 M	4
10X PBS	10X	1
Water		0.9
**Total**		**9.4**

**Table 3:** ExM empty gel and peptide-containing gelation solutions

Reagent	Concentration	Final concentration	Add
StockX monomer solution			60 μL
Additional BIS (not including concentration of BIS in the StockX solution)	130 mM (2% (w/v))	13 mM	10 μL
Peptide (replace with water for the empty gel gelation solution)	5 mM	1 mM	20 μL
APS	10% (w/v)	0.5% (w/v)	5 μL
TEMED	10% (v/v)	0.5% (v/v)	5 μL
Total volume			100 μL
